# Spectrum of Protein Location in Proteomes Captures Evolutionary Relationship Between Species

**DOI:** 10.1007/s00239-021-10022-4

**Published:** 2021-07-30

**Authors:** Valérie Marot-Lassauzaie, Tatyana Goldberg, Jose Juan Almagro Armenteros, Henrik Nielsen, Burkhard Rost

**Affiliations:** 1 Department of Informatics & Center for Bioinformatics & Computational Biology – i12, Technical University of Munich (TUM), Boltzmannstr. 3, 85748 Garching/Munich, Germany; 2 Institute for Advanced Study (TUM-IAS), Lichtenbergstr. 2a, 85748 Garching/Munich, Germany; 3 TUM School of Life Sciences Weihenstephan (WZW), Alte Akademie 8, Freising, Germany; 4 Department of Biochemistry and Molecular Biophysics, Columbia University, 701 West, 168th Street, New York, NY 10032 USA; 5 Novo Nordisk Foundation Center for Protein Research, Faculty of Health and Medical Sciences, University of Copenhagen, 2200 Copenhagen N, Denmark; 6 Section for Bioinformatics, Department of Health Technology, Technical University of Denmark, 2800 Kgs. Lyngby, Denmark

**Keywords:** Genome sequence analysis, Protein location, Species comparisons, Prediction of cellular compartment

## Abstract

The native subcellular location (also referred to as localization or cellular compartment) of a protein is the one in which it acts most frequently; it is one aspect of protein function. Do ten eukaryotic model organisms differ in their *location spectrum*, i.e., the fraction of its proteome in each of seven major cellular compartments? As experimental annotations of locations remain biased and incomplete, we need prediction methods to answer this question. After systematic bias corrections, the complete but faulty prediction methods appeared to be more appropriate to compare location spectra between species than the incomplete more accurate experimental data. This work compared the location spectra for ten eukaryotes: *Homo sapiens* (human), *Gorilla gorilla* (gorilla), *Pan troglodytes* (chimpanzee), *Mus musculus* (mouse), *Rattus norvegicus* (rat), *Drosophila melanogaster* (fruit/vinegar fly), *Anopheles gambiae* (African malaria mosquito), *Caenorhabitis elegans* (nematode), *Saccharomyces cerevisiae* (baker’s yeast), and *Schizosaccharomyces pombe* (fission yeast). The two largest classes were predicted to be the nucleus and the cytoplasm together accounting for 47–62% of all proteins, while 7–21% of the proteins were predicted in the plasma membrane and 4–15% to be secreted. Overall, the predicted location spectra were largely similar. However, in detail, the differences sufficed to plot trees (UPGMA) and 2D (PCA) maps relating the ten organisms using a simple Euclidean distance in seven states (location classes). The relations based on the simple predicted location spectra captured aspects of cross-species comparisons usually revealed only by much more detailed evolutionary comparisons. Most interestingly, known phylogenetic relations were reproduced better by paralog-only than by ortholog-only trees.

**Supplementary Information:**

The online version contains supplementary material available at 10.1007/s00239-021-10022-4.

## Introduction

### Location Spectrum of an Organism

Eukaryotic cells contain many distinct compartments separated by membranes. This separation allows to create functionally specialized spaces with slightly different biophysical features (Alberts 2002). Therefore, the atlas of where proteins predominantly perform their function—their native location or compartment—contains important information about protein function that is used to classify function in the Gene Ontology (GO) Consortium (Ashburner et al. 2000) in terms of what is referred to as cellular compartment (here, we used the more commonly used term *location*, instead). While usually only the location of a particular protein of interest matters, our analysis began from two simple academic questions. First, do we have enough information to describe location for all proteins in entire organisms? We refer to the *location spectrum* as to the percentage of proteins in each location for an entire organism; one component of this spectrum is, e.g., the fraction of secreted proteins. Second, if we do, does anything as simple and abstract as the location spectrum contain any relevant information about an organism? If so, this might ultimately help to spot sets of proteins most relevant for functional shifts between two organisms.

### Can a Phylogenetic Tree Be Inferred from the Location Spectrum?

Zuckerkandl and Pauling initiated an ongoing debate about the molecular constituents of evolution (Zuckerkandl and Pauling 1965). The location spectrum is determined by the sequences of the proteins forming the proteome, and as such it is based on tertiary semantides in the sense of Zuckerkandl and Pauling. However, the location spectrum projects the complex information captured by the amino acid sequence onto a low-dimensional composition vector that—unlike the sequence—is not limited to discrete states. Thus, we might attempt reconstructing a phylogenetic tree from location spectra through the pairwise distances between the composition vectors. Such an approach somehow mimics the immunological precipitin reaction, which formed the basis of the earliest attempts to use molecules for phylogenetic classification (Suárez-Díaz 2016). Due to the extreme information reduction from thousands of sequences to a small number of dimensions (the number of locations under analysis), we found it quite remarkable that the location spectra captured aspects of higher-level taxonomy.

### Most Proteins Have One Dominant “Native” Location

Many proteins “travel,” i.e., they function in more than one location over the course of their existence. Most proteins, however, have one predominant, native location to accomplish their function as suggested by the following argument. Assume the opposite, namely that each protein is equally frequently observed in *D* different locations. If we forced annotations to be limited to a single location (i.e., ignore all annotations we find other than the first) and we compared two sets of annotations of location (from predictions or experiments) that are essentially error free, comparing 1000 proteins, we would find 1000/*D* to agree between the methods due to picking only one of *D*. Some analysis of data for human might be misunderstood to suggest *D* = 3 (Results). For this number, 1000/3 = 333 proteins would agree due to the combination of error-free annotations and picking only the first annotation. Assuming *L* classes of location (for simplicity let *L* be 10), then one tenth of the 667 disagreeing (1000-333) proteins would match at random, i.e., another 67, bringing the total to 400. In other words, the agreement between two error-free datasets would appear to be 40%. For *D* = 2 (two native location per protein), this number would rise to 55%, for *D* = 1.5 to 78%. However, good prediction methods reach levels of performance above 65% in 18 states, and above 80% for the best classes (Goldberg et al. 2012) even when using an annotation dataset that assumes only one location to be correct. Thus, this little back-of-an-envelope calculation refutes the hypothesis that most proteins have more than one “native” location.

### Annotating Location in Human Proteome

We first asked to which extent the assembly of all experimental data provides a good approximation toward describing the *location spectrum* of an organism. Previous experimental studies have attempted to determine the proteome-wide location spectrum for model organisms such as baker’s yeast (*Saccharomyces cerevisiae*) (Huh et al. 2003) and *Pseudomonas aeruginosa* (Lecoutere et al. 2012). Despite almost two decades of such successful studies, “only” 75% of all yeast proteins are classified into one of 22 locations. This lack of completeness in coverage is one limitation for using experimental data to compute the location spectrum. The other pertains to the issue of “travelers” vs. “natives”: most contemporary experiments pick up all locations in which proteins have been observed as opposed to the most frequent or native location. To use a simple analogy, we cannot derive the country in which people live from the list of countries they visit. 

A more complex organism with many readily available experimental location annotations is *Homo sapiens*. Assuming a predominant native location for each protein, we analyzed the agreement between experimental results, then combined results from Swiss-Prot (Boutet et al. 2016) and the Human Protein Atlas (HPA) (Thul et al. 2017) into a large set with reliable annotations, and analyzed how much the given experimental annotations reveal about the expected location spectrum in these two organisms. Less accurate predictions of the native location available for all proteins from prediction methods provided a much better proxy for the real location spectrum of an organism than much more accurate but incomplete experimental observations unable to differentiate native and non-native locations (SOM: Supporting Online Materials Fig. S4 and S5 cf. Results). In order to really establish this result, we needed to correct the bias introduced by prediction methods that predict some locations better than others (Marot-Lassauzaie et al. 2018). Given this error correction, we predicted the location spectrum for ten model organisms. As expected, organisms with similar proteins had similar location spectra. Surprisingly, the differences sufficed to reproduce some aspects of the phylogenetic relationships between the organisms. This finding indicates that the location spectrum contains relevant information specific to each organism.

## Methods and Materials

### Proteomes

The sequences for the reference proteomes were taken from the EMBL-EBI database (https://www.ebi.ac.uk/reference_proteomes) (Altenhoff et al. 2016). The Human proteome was taken from the 4th release of 2016 which contains 21,018 proteins. For the cross-species comparison, nine reference organisms were chosen from release 2017-4. The ten proteomes were human (*Homo sapiens*), chimpanzee (*Pan troglodytes*), gorilla (*Gorilla gorilla*), mouse (*Mus musculus*), rat (*Rattus norvegicus*), fruit fly (*Drosophila melanogaster*), African malaria mosquito (*Anopheles gambiae*), nematode (*Caenorhabditis elegans*), baker’s yeast (*Saccharomyces cerevisiae*), and fission yeast (*Schizosaccharomyces pombe*).

### Experimental Annotations

Experimental annotations for the location of human proteins were taken from Swiss-Prot (Boutet et al. 2016) and The Human Protein Atlas (HPA) (Thul et al. 2017). This resulted in a set of 5563 proteins with experimental annotations from Swiss-Prot (release 2017_1; human reference proteome up000005640), and in 12,036 from The Human Protein Atlas (version 15; confined to 32 location classes). We had access to one additional set of experimental annotation in GO format extracted from scientific literature by the tool LocText (Cejuela et al. 2018).

To infer the location spectra through homology-based inference (HBI, below) for the ten reference organisms, we extracted all proteins with verified experimental annotation from Swiss-Prot (release 2021_01; evidence code ECO:0000269). This resulted in a reference setExp of 34,861 proteins (without redundancy reduction).

For this analysis of the agreement between HPA and Swiss-Prot, we only considered the subset of the proteins for which both Swiss-Prot and HPA had experimental annotations. Annotations were counted as identical when any location matched, e.g., a protein with the Swiss-Prot annotation “nucleus, cytoplasm” and the HPA annotation “cytoplasm, mitochondria, Golgi” was considered to have identical annotations.

### Prediction Methods

The following six prediction methods were compared for human. LocTree2 (Goldberg et al. 2012) uses profile kernels Support Vector Machines (SVMs) through a decision tree; it predicts one of 18 locations for eukaryotes. LocTree3 (Goldberg et al. 2014) combines LocTree2 with homology-based inference (in as many different classes as are experimentally annotated). Hum-mPloc3.0 (Zhou et al. 2017) predicts 12 location classes by combining residue-based statistical features, with conserved domains and Gene Ontology annotations. Several classes are predicted for each protein; only the one with the highest score was kept. MultiLoc2 (Blum et al. 2009) predicts 11 location classes by integrating the output of six sequence-based classifiers (SVMTarget, SVMSA, SVMaac, MotifSearch, GOLoc, PhyloLoc) through a final SVM. WoLF PSort (Horton et al. 2007) predicts 10 location classes by first converting a sequence into features indicative of location (amino acid composition, sorting signals, and functional motifs). A k-nearest neighbor classifier is applied to those features to predict. DeepLoc (Almagro Armenteros et al. 2017) uses deep neural network to assign proteins to one of 10 subcellular locations.

The six prediction methods were evaluated against two reference sets of proteins with known annotations containing many proteins that had most likely not been used to develop and assess the prediction methods applied. The first reference set was taken from the HPA (published after the development of the methods). The second reference set was extracted from scientific publications using LocText (Cejuela et al. 2018). None of those had been annotated in Swiss-Prot. To avoid further complications, only proteins with a single annotation were kept. This resulted in a set of 2000 proteins with reliable experimental annotations from HPA and 1315 with less reliable maps to annotations from LocText (less reliable due to possible mistakes in the text mining).

Prediction methods differ in their level of detail; to simplify the comparison, we projected all predictions onto seven main location classes shared between all tools (SOM: Table S1 for mapping): secreted, nucleus, cytoplasm, plasma membrane, mitochondrion, endoplasmic reticulum (ER), and Golgi apparatus. All proteins for which the experimental annotations could not be mapped to those seven classes were excluded from the analysis.

### Error Corrections for Estimates of Distributions

Prediction methods tend to make specific mistakes, and these mistakes are not the same for all classes. Therefore, the predicted spectrum of locations for an entire organism cannot be estimated accurately enough from using the output of prediction methods directly. Instead, the specific errors have to be corrected (Marot-Lassauzaie et al. 2018). Toward this end, the confusion matrices for each prediction method needed for the correction were built by establishing the performance for human proteins with a single experimental annotation in Swiss-Prot.

### Error-Correction Using Confusion Matrix

As described previously (Marot-Lassauzaie et al. 2018), we corrected the predicted location spectra through the performance estimates given in the confusion matrix *M*_*p,o*_ (TP true positives, TN true negatives, FP false positives, and FN false negatives), where the elements *M*_*p,o*_ give the number of proteins predicted in state *p* and observed in state *o*. The diagonal contains the correct predictions with *p* = *o*. From *M*, we can compute a new n*n matrix *M'* with

1 $$M{{^{\prime}}}_{\left(p,o\right)}={M}_{\left(p,o\right)}/\left({\sum }_{\left(i=1\right)}^{n}{M}_{\left(p,i\right)}\right).$$

This new matrix provided the ratio by which each location was normalized. The predictions for an entire dataset *P* = *(p*_*1*_*,p*_*2*_*,…,p*_*n*_), where *p*_*i*_ is the ratio of the proteins of the dataset predicted to be in state *i*, were corrected to *C* = *(c*_*1*_*,c*_*2*_*,…,c*_*n*_*)* with ${c}_{x}={\sum }_{\left(i=1\right)}^{n}{M}_{\left(i,x\right)}^{{^{\prime}}}*{p}_{i}.$.

### Distance Between Distributions

For human proteins, the 7-state distributions directly obtained from the experimental annotations were compared to those predicted by the five selected prediction tools. The straightforward Euclidean distance for this 7-state distribution served as proxy for the difference between predictions and experiments. The distance *d* between two points *p* = *(p*_*1*_*,p*_*2*_*,…,p*_*n*_*)* and *q* = *(q*_*1*_*,q*_*2*_*,…,q*_*n*_*)* in a n-dimensional state is given by the Pythagorean formula:

2 $$d\left(p,q\right)=\sqrt{\left({\sum }_{\left(i=1\right)}^{n}{\left({q}_{i}-{p}_{i} \right)}^{2}\right)}.$$

Here, the seven dimensions were given by the location spectrum in seven main subcellular location classes. For the other nine model organisms, the same Euclidean distance established how similar those organisms were according to the predicted spectrum of locations. These distances were visualized in two different ways: first, through an unweighted pair group method with arithmetic mean (UPGMA) (Michener 1958) tree built using the R-package phangorn (Schliep 2011) and second, through a 2D view originating from principal component analysis (PCA) representation of the distances with the stats R-package (R Core Team 2017).

### Clustering Proteins into Families

To identify proteins with very similar sequences, we grouped all protein pairs with small HSSP value, more precisely with HVAL < 4; (Mika and Rost 2003). The HVAL is the distance from the HSSP curve (Rost 1999; Sander and Schneider 1994) (+ above curve; − below) which combines alignment length (*L*) and percentage pairwise sequence identity (*PID*; 1 if identical amino acid, 0 else; gaps not counted) to empirically distinguish between proteins with similar structure (Rost 1999; Sander and Schneider 1994), location (Nair and Rost 2002), or enzymatic activity (Rost 2002). For alignments longer than 250, HVAL = 0 implies about 20% *PID*; more explicitly the value is defined as follows (Mika and Rost 2003):

3 $${\text{HVAL}}\left( {L, PID} \right) = PID - \left\{ {\begin{array}{ll} {100} & {{\text{for}}\, L \le 11} \\ {480 \times L^{{ - 0.32 \times \left[ {1 + \exp \left( { - L/1000} \right)} \right]}} } & {{\text{for}} \,L \le 450} \\ {19.5} & {{\text{for}} \,L > 450} \\ \end{array} } \right.$$

where *L* is the alignment length (number of residues for which two proteins are aligned, not counting insertions and deletions), and *PID* is the number of identical residues in the alignment times 100 and divided by *L*.

Pairs of proteins with similar sequences tend to have similar native locations (Nair and Rost 2002). For instance, over 92% of the sequence similar pairs of proteins have the same location (Nair and Rost 2002) at an HVAL > 4 (Eq. 3) when comparing a set of proteins native to just four locations (nucleus, extracellular space, cytoplasm, and mitochondria). We clustered all human proteins at this threshold using *UniqueProt* (Mika and Rost 2003). All 21,018 human proteins grouped into 3148 *families* (defined as HVAL > 4 for all proteins in that *family* to a representative seed protein). These families were roughly chosen such that all members of one family likely share their native location (Methods (Nair and Rost 2002)).

### Homology-Based Inference of Location (HBI)

Our simple approach toward homology-based inference of location proceeded as follows. For a query protein sequence Q, Q is aligned to all proteins of experimentally known location (setExp). If the closest hit in setExp was more similar than a certain threshold, we copied the location for this closest hit to Q. MMSeq2 (Steinegger and Söding 2017) queried each sequence of the ten proteomes against setExp with 34,861 proteins. We set the sensitivity of MMSeq2 to 7.5 (highest possible sensitivity) and only reported matches with an e-value smaller than 1.

### Location Spectra For in-Paralogs and Orthologs

Sets of in-paralogs and ortholog groups for the ten reference organisms were extracted from InParanoid (Sonnhammer and Östlund 2015). Proteins with similar sequence arising from a speciation event are referred to as orthologs, and those arising from gene duplication are referred to as paralogs. We further differentiated between gene duplication predating speciation (dubbed out-paralogs) possibly present in both species, and gene duplication postdating speciation (dubbed in-paralogs). In-paralogs can be co-orthologs to orthologs in the other species.

The subset of genes found to be orthologs between species but that were not in an in-paralog group in comparison to any other species was used to evaluate the effect of sequence deviation over time on the changes in location spectra. The subset of in-paralogs between any pair of species, without the most likely ancestor gene, taken as the ortholog used to identify this group, was used to evaluate the effect of paralogs on the changes in location spectra.

## Results and Discussion

Our main result was that the percentage of proteins native to each of ten major location classes in entire proteomes (referred to as the *location spectrum*) captures phylogenetic relationships between organisms. To show this required the establishment of two other results. First, that while experimental data for protein location are essential for the annotation of entire proteomes, those data remain insufficient (available for too few proteins) and too biased (over-representation of some location classes). Second, results from error- and bias-corrected prediction methods apparently predicted location spectra accurately. Given these two results, location spectra can be compared adequately between organisms only based on predicted values.

### Experimental Data Essential to Annotate Yeast and Human Proteome but Insufficient to Gage Location Spectrum

#### Reliable Experimental Location Annotations for 37% of All Human Proteins

**Fig. 1:**
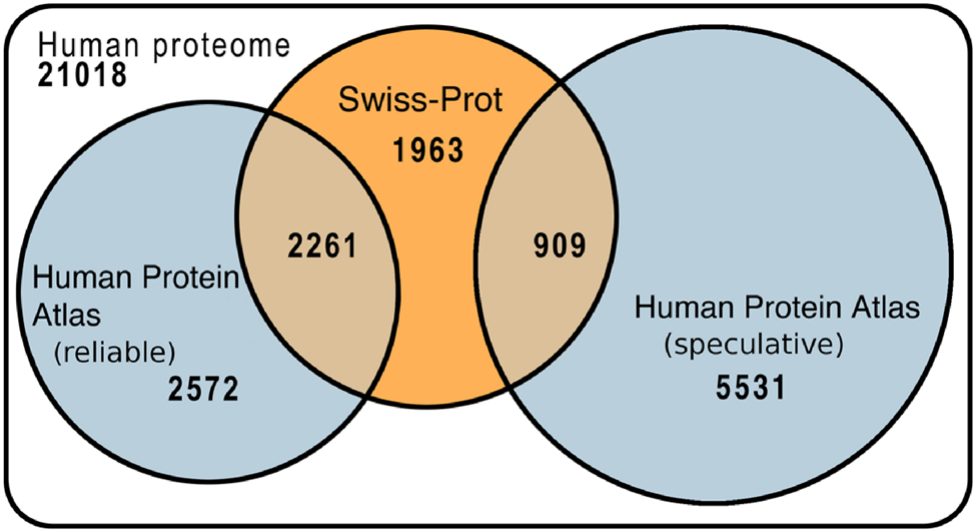
Protein location in human proteome annotated by experiments. The Venn diagram compares experimental annotations of human proteins between Swiss-Prot (Boutet et al. 2016) and The Human Protein Atlas (HPA) (Thul et al. 2017). The white background (all 21,018 human proteins) is not to scale. We grouped the four HPA annotation levels into *reliable* (94% agreement with Swiss-Prot, Table 1) and *speculative* (54% agreement with Swiss-Prot only slightly above random, Table 1). For instance, 2261 proteins have HPA reliable annotations and match at least one Swiss-Prot annotation (evidence code: ECO:0000269), while 37% of all human proteins (7705 = 1963 + 2572 + 2261 + 909) have reliable experimental annotations (Swiss-Prot ECO:0000269 or HPA *validated* and *supportive*)

Both Swiss-Prot (Boutet et al. 2016) and the Human Protein Atlas (HPA) (Thul et al. 2017) experimentally annotate subcellular location of proteins. HPA exclusively uses experimental annotations of varying quality (read “ > ” as “better than”: Validated > Supportive > Uncertain > Unreliable); Swiss-Prot (Boutet et al. 2016) evidence code ECO:0000269 also focuses on experimental data. If both databases were error free, their annotations would fully agree (Methods). Indeed, 94% of the HPA annotations with higher reliability (HPA level: *Validated* and *Supportive*: 2261 cases, Table 1) agreed with Swiss-Prot, while only 54% of those with lower reliability did (HPA level: *Uncertain* and *Unreliable*: 909 cases; Table 1; Fig. 1). Considering the first value (94%) high enough to label those annotations as “*reliable”* (others as “*speculative”*; Table 1), 37% of the human proteins have reliable experimental information (7705, Fig. 1). If we estimated the *location spectrum* with those 37%, we would end up with two estimates: (1) proteins would, on average, be in 1.3 location classes (SOM: Fig. S4A); (2) the location spectrum estimated for the entire proteome would appear non-sense, with nearly half of the proteome (46%) as nuclear (Fig. S4A: orange track), 41% as cytoplasmic (Fig. S4A: red track), and very few secreted proteins as this class is missing in HPA and underrepresented in Swiss-Prot. Another organism with many experimental location annotations is baker’s yeast (*Saccharomyces cerevisiae*): 43% of all yeast proteins have experimental location annotation in Swiss-Prot. Similar to the situation for human, if we generalized from yeast, a protein would appear, on average, in 1.2 location classes (SOM Fig. S5A), and most proteins would incorrectly appear to be either nuclear (41% Fig. S5A: orange track) or cytoplasmic (42% Fig. S5A: red track).

**Table 1:** Reliable Human Protein Atlas (HPA) annotations agree with Swiss-Prot

HPA level	Nprot	HPA = Swiss-Prot (%)	Expected (%)
Validated	644	95	38
Supportive	1617	94	39
Uncertain	783	57	45
Unreliable	126	39	44
Merged			
Reliable	2261	94	39
Speculative	909	54	45

*HPA level* reliability provided by Human Protein Atlas (HPA) (Thul et al. 2017), *Nprot* number of human proteins compared (only HPA proteins with Swiss-Prot match), *HPA* = *Swiss-Prot* percentage of proteins for which any annotation in Swiss-Prot (experimental only Boutet et al. (2016)) agrees with at least one annotation in HPA, *Expected* agreement between annotations after random shuffle (randomly pick proteins from Swiss-Prot set, compare to HPA proteins of corresponding HPA level, repeat 100 times for each level and average), *Merged* the two best HPA levels (high agreement to Swiss-Prot) were merged into “reliable”, the two worst into “speculative”

### Location Reliably Inferred for 62% of the Human Protein Families

1920 (62%) human and 1144 (66%) yeast protein families were covered by experimental annotation from at least one protein within the family (Methods). As those families tended to be larger than average, homology-based inference covered 89% of the human proteins (18,840) and 84% of the yeast proteome (5110).

Despite this high coverage, homology-based inference alone could not estimate the full *location spectrum* in an organism as explained by the following argument. If we used the combined annotations of both databases to infer location for the families, the number of multiple locations would be very high: 16,378 of the 18,840 human proteins and 3760 of the 5110 yeast proteins were predicted by homology in multiple compartments (on average 3.3 compartments per protein for human and 2.2 for yeast). If true, our comparisons between Swiss-Prot and HPA could not have reached above 40% agreement (argument in 2nd paragraph of Introduction). In contrast, the top HPA annotations (class: *reliable*) reached 94% (Table 1) suggesting the implied fraction of 3.3 compartments per protein to be incorrect by more than a factor of two. Another non-sense consequence was the following: if we used only experimental and homology-inferred annotations, we would predict 15,169 human proteins (72%) to be nuclear (corresponding to 68% of the families; SOM: Fig. S4). Another value that might be too high by a factor of two. Even more misleading was the implication that 49% of all human protein families implied with crossing the plasma membrane (SOM: Fig. S4) compared to fewer than 25% of all human proteins predicted to have at least one transmembrane helix inserted into membranes of any compartment (Bernhofer et al. 2016). Homology-based inference of location (Methods) for yeast produced similar non-sense results, with 81% and 82% of the proteins (corresponding to 66% and 66% of the families; SOM: Fig. S5) predicted to be nuclear or cytoplasmic, respectively. Another stark over-estimate was considering 39% of all yeast proteins as mitochondrial. In contrast, only 7% of the yeast families were found to be associated with the plasma membrane. Comparing the number to that for human (49%), it is obvious that both numbers are likely off more than twofold.

Homology-based inference of location spectra from proteins with experimental knowledge in Swiss-Prot for a set of reference organisms also largely failed to capture the differences between species (SOM: Fig S10). Thus, the spectrum of locations for an organism cannot be inferred from experimental and homology-inferred annotations alone.

### Location Spectra Accurately Predicted

Given that the location spectrum cannot be established through experimental annotations alone, not even for model organisms as well covered as *Homo sapiens* and *Saccharomyces cerevisiae*, the problem became: how to establish that prediction methods could succeed where experimental data fail. We assembled two new data sets, both containing proteins that had not been used for the development of any of the methods tested. The first set assembled all reliable HPA annotations not overlapping with Swiss-Prot (all methods used Swiss-Prot annotations for development), dubbed *HPA_subset*. The second was generated by automated text mining (Cejuela et al. 2018) and also contained only proteins not in Swiss-Prot, dubbed *LocText_subset*. With those two subsets, we assessed how well the location spectrum of an entire set was predicted to proxy the “true” experimental distribution in an entire proteome. Although both those sets were subject to experimental bias clearly not reflecting entire proteomes, crucially, the two were compiled with very different types of bias.

#### Error Corrections Remove Bias and Capture Location Spectrum for Organisms

Overall, most prediction methods came out-of-the-box close to estimating the 7-class *location spectrum* for human proteins (SOM: Fig. S6 left). When error correcting the predicted spectra according to the performance confusion matrix (Marot-Lassauzaie et al. 2018), the observations and predictions became even more similar for most methods (Fig. S6 right). After error correction, Hum-mPloc3.0 estimated the location spectrum of the experimental data best. However, since this tool is limited to human, we had to continue our cross-organism analysis with the second and third best solutions, namely the error-corrected version of DeepLoc and LocTree3.

#### Location Spectra for Ten Model Organisms Predicted to be Similar

Predictions of the 7-location spectra for ten completely sequenced model eukaryotes were computed by DeepLoc and LocTree3 (Fig. 3, SOM: Table S2 and S3). The predicted location spectra were largely similar between the ten proteomes.

For all ten model organisms investigated, nucleus and the cytoplasm were the largest location classes (highest fraction of proteins; Fig. 3, right panel, SOM: Fig. S9). The highest fraction for those two was for fission yeast (*Schizosaccharomyces pombe*; 62%), the lowest for the nematode (*Caenorhabditis elegans*; 47%). The largest relative difference between the ten was predicted for the extracellular space (*secreted* in Fig. 3) with fission yeast as the lowest (4%) and the African malaria mosquito (*Anopheles gambiae*)/nematode as the highest (15%). Runner up in terms of difference was the Endoplasmic reticulum (ER) with baker’s yeast (*Saccharomyces cerevisiae*)/fission yeast as top (16%) and mouse (*Mus musculus*) as the lowest (6%). The fraction of proteins predicted in the plasma membrane spanning from 7% (fission yeast) to 21% (mouse/rat—*Rattus norvegicus*) was much more compatible with methods predicting helical membrane proteins than estimates extrapolated from experimental annotations. The same appeared the case for other classes (in particular: for nucleus, mitochondria, and ER).

##### Location Spectra Capture Evolutionary Relationship Between Species

**Fig. 2:**
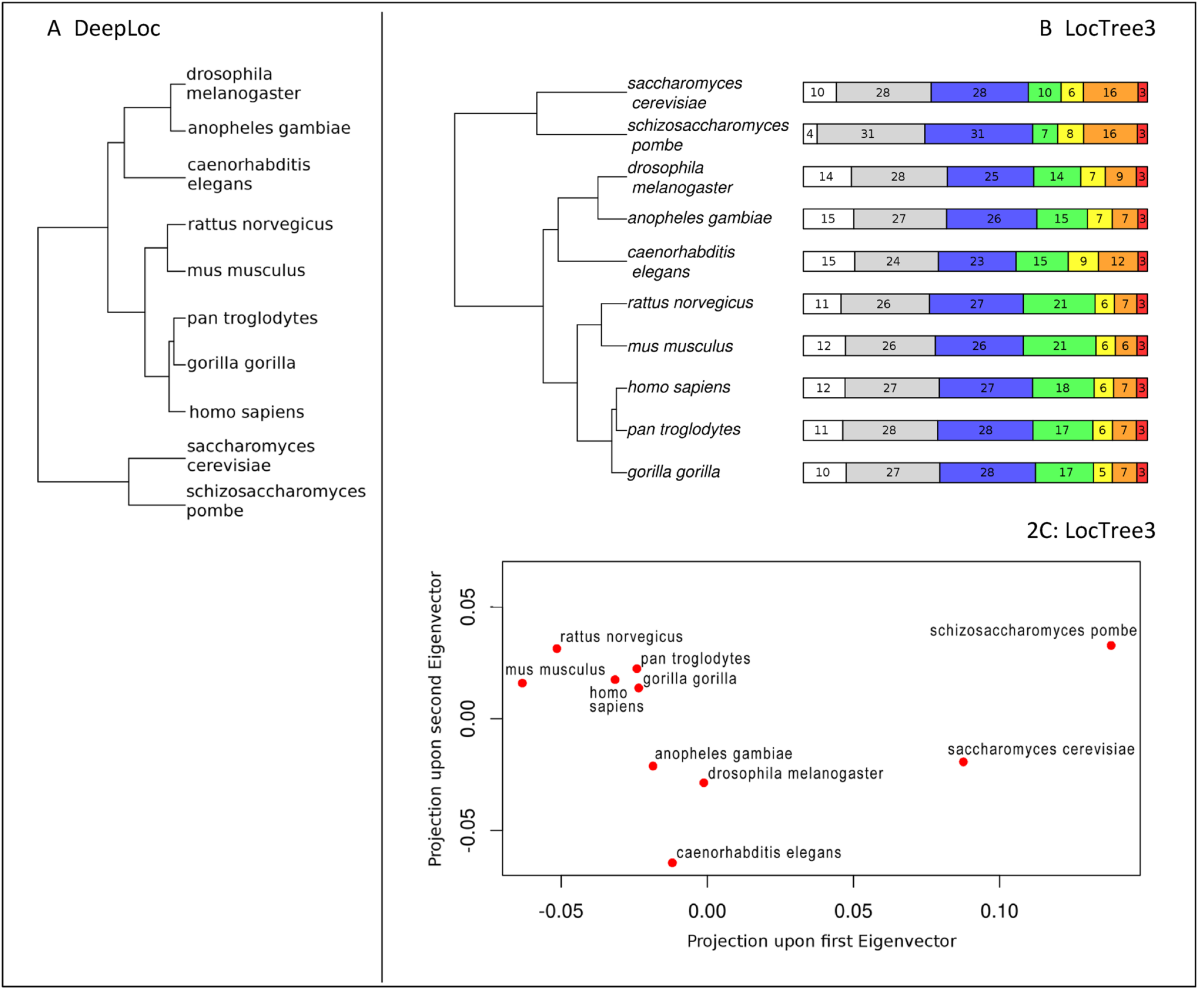
Grouping of ten eukaryotes according to predicted *location spectra*. We computed the Euclidean distances (Eq. 2) between the proteome-wide distributions predicted by LocTree3 with error correction (Eq. 1) (Marot-Lassauzaie et al. 2018) for each of the ten reference organisms. Those values were plotted onto a UPGMA tree (top panel **A**) and shown through PCA in 2D (lower panel **B**). **A** UPGMA tree built from the predicted distributions from DeepLoc for the 10 organisms. **B** UPGMA tree along with a bar representing the predicted distribution from LocTree3 in the seven main subcellular location classes is shown for each organism. The seven location classes (from left to right): secreted (white), nuclear (gray), cytoplasmic (blue), plasma membrane (green), mitochondrial (yellow), endoplasmic reticulum (orange), and Golgi apparatus (red). Despite the small differences, the resulting tree largely agrees with what we expect from evolution. **C** The PCA adds more details to the comparison between species from the LocTree3 predictions. Two interesting aspects are the large differences between the two yeast species (*y*-axis) and the approximate triangle between mouse, rat, and human

The small but significant differences in the location spectra predicted between the ten model organisms sufficed to draw UPGMA-trees relating those organisms that appeared reasonable in the following key aspects (Fig. 2AB, SOM: Fig. S7A). (1) The two yeast types were grouped together and separated from the multicellular organisms. (2) Mammals were grouped together. (3) The two rodents were separated from the three apes. (4) According to the tree originating from LocTree3, chimpanzee (*Pan troglodytes*) and human (*Homo sapiens*) were considered to be closer to each other than either was to gorilla (*Gorilla gorilla*; Fig. 2B). This last result was not reproduced using DeepLoc predictions (detailed discussion below).

Representing the relations from a matrix (of O × 7 dimensions for 7 major locations and O organisms) giving the location spectrum through a one-dimensional tree necessarily simplifies the relations contained in the data (matrix) and thereby might miss important relations. Therefore, for the LocTree3 predictions, we also displayed the relations in two dimensions (Fig. 2D, SOM: Fig. S7B) through a simple principal component analysis (PCA; first two eigenvector projections shown in Fig. 2C). The 2D view confirmed the principal findings from the tree and added interesting details. For instance, in 2D, the two yeast types remain separated from all multicellular organisms but are much more separated from each other on the *y*-axis (2nd eigenvector) than for instance the apes from each other. This could be due to the smaller size of their genome comparatively to the other organisms or to the short generation time which allows for a faster divergence. Another interesting observation was that the proximity on the y-axis (2nd eigenvector) between both rodents (mouse and rat) was similar to the proximity of each rodent to human. Here again, this observation could be explained by the shorter generation time of these organisms allowing for a comparatively high distance.

**Fig. 3:**
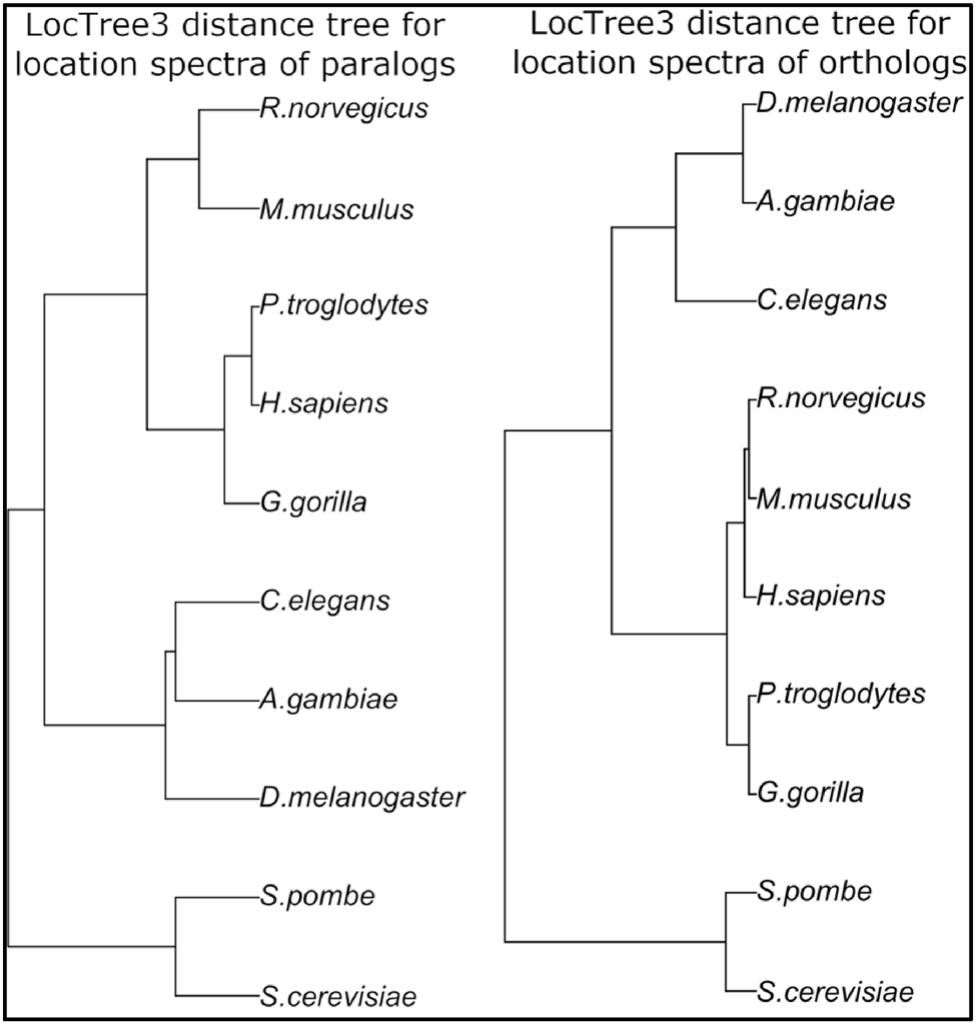
Grouping of ten eukaryotes according to location spectra of paralogs and orthologs only. We used InPAranoid to identify all in-paralogs and orthologs for the ten species, and extracted the LocTree3 prediction for these two subset of genes. We computed the Euclidean distances (Eq. 2) between the predicted distributions predicted by LocTree3 (without correction) and used these distances to build a UPGMA tree for each subset of genes. The mean distance of location spectra for paralogs is two times greater for paralogs than for orthologs, but the trees are scaled to visually remove this effect

Two main factors drive the divergence of location spectra between species. On the one hand, the sequence of orthologous proteins can diverge between species to the point at which location changes. On the other hand, gene duplication events happening after speciation will shift the location spectrum unless all duplications are done in exactly the same frequency as the spectrum. For both LocTree3 and DeepLoc, the predicted locations of paralogs agreed in over 90% of the cases, indicating that gene duplication events mostly maintain the location spectrum. To gage the strength of these effects, we extracted only the subset of genes that come from gene duplication events after speciation (*in-paralogs*). We also extracted the subset of orthologs without these in-paralogs. Comparing the predicted location spectra of these two subsets of genes (Fig. 3, SOM: Fig. S11) shows that the mean distance of in-paralog spectra was twice that for orthologs. Both for DeepLoc and LocTree3, the trees for paralogs were closer to the trees expected from evolution. In contrast to the situation for all proteins, the in-paralog tree from DeepLoc was now fully correct (Fig. S11B), while that for LocTree3 (Fig. 3) misclassified *A. gambiae* to be closer to *C. elegans* than to the other insect (*D. melanogaster*).

One interpretation is to consider the differences between the trees implied by predictions from LocTree3 and DeepLoc as “error bars.” In this view, the tree implied by LocTree3 mapping the evolution of the triangle human, chimp, gorilla better than that for DeepLoc would simply be “good luck” for LocTree3. Given that DeepLoc slightly outperformed LocTree3 (Fig. S6), we assume that for 1000 other triangles, resulting from sampling another 10,000 (10 k) organisms, this “luck” would reverse more than 500 times. Indeed, the branches separating gorilla from chimp and human for LocTree3 or human from chimp and gorilla for DeepLoc are very short, and the difference could be within the noise of the prediction. Conversely, the newer DeepLoc might have used proteins for development that were treated implicitly as “independent test cases.” If so, this might suggest slight over-estimations in the values for DeepLoc in SOM: Fig. S6 (and possibly those for other methods newer than LocTree3). If so, LocTree3 might generate a better tree. In this view, the difference would indicate how small differences in performance might have detectable effects on the interpretation of the spectrum of locations.

According to the concept of a molecular evolutionary clock introduced by Zuckerkandl and Pauling (Zuckerkandl 1987; Zuckerkandl and Pauling 1962), molecular traits evolving in a continuous, “neutral” manner over time proxy the time of divergence of species better than characters evolving irregularly (Morgan 1998). For molecular traits under functional selective pressure, the rates of divergence tend to vary over time. Hence, it was not a priori clear that the protein location spectrum could correctly estimate evolutionary distances. However, as long as the variation in the rate of evolution is smaller than the evolutionary distances compared, such molecular traits could succeed in estimating inter-species distances. In this perspective, the differences between expert-based and location spectrum-based trees might be explained as a limit in the resolution of this approach.

Whichever of those views will turn out to be closer to the truth, clearly both solutions provided trees that given the degree of simplicity suggest a stunning similarity between the evolution of species and the spectrum of locations. In fact, another indication for relative robustness of the tree was the observation that the trees generated by DeepLoc and LocTree3 before and after error correction were similar (SOM: Fig. S8 C and D). When method performance falls below an unspecifiable threshold, trees before and after error correction change substantially (SOM: Fig. S8A).

## Conclusions

We showed that experiments enriched by homology-based inference accurately annotate the subcellular location for 89% of all human proteins (Fig. 1). Nevertheless, the location spectrum for entire proteomes, i.e., the composition of proteins in each of the major compartments appears to be estimated rather incorrectly by annotations exclusively from experiments or from experimental data enriched by homology-based inference (SOM: Fig. S4, S5, and S10). In contrast, prediction methods reliably estimate location spectra after correcting for prediction mistakes and experimental bias (SOM: Fig. S6). Applying such an error-corrected whole-proteome prediction with DeepLoc and LocTree3 to ten model organisms suggested rather similar location spectra (Fig. 2). Yet, the small differences sufficed to build a tree based on the location spectra that reflected aspects of the evolution between the ten model organisms both on the level of a 1D tree (Fig. 2AB) and of a 2D proximity map (Fig. 2C). These findings might suggest that changes to the location spectrum are one mechanism to evolve new functions on the level of species.

## Supplementary Information

Below is the link to the electronic supplementary material.

Supplementary file1 (DOC 6843 kb)

## Data Availability

Data and source code associated with this work are available at ftp://rostlab.org/locspecies/data.zip

## Abbreviations

3D: Three dimensional
3D structure: Three-dimensional coordinates of protein structure
ER: Endoplasmic reticulum
GO: Gene ontology
HBI: Homology-based inference
HPA: Human protein atlas
HVAL: HSSP value
PCA: Principal component analysis
PID: Pairwise sequence identity
SOM: Supporting Online Materials
UPGMA: Unweighted pair group method with arithmetic mean
