# Supplementary material for: Spectrum of Protein Location in Proteomes Captures Evolutionary Relationship Between Species
Source: J Mol Evol. 2021 Jul 30;89(8):544–53. doi: 10.1007/s00239-021-10022-4 (PMC8379119; doi:10.1007/s00239-021-10022-4)
Supplement: Supplementary file 1 — Supplementary file1 (DOC 6843 kb) [file 239_2021_10022_MOESM1_ESM.doc]

*Supporting online material (SOM) for:*Spectrum of protein location in proteomes captures evolutionary relationship between species

Valérie Marot-Lassauzaie, Tatyana Goldberg, Jose Juan Almagro Armenteros, Henrik Nielsen & Burkhard Rost

# Short description of SOM

This file contains the supporting online material (SOM) for the publication “Spectrum of protein location in proteomes captures evolutionary relation between species”.

• **Table S1** shows how the output from the three prediction tools and from The Human Protein Atlas (Thul et al. 2017) was converted to a set of seven main location classes for the comparison of the output.

• **Table S2** gives the error-corrected number of proteins predicted in each of the seven location classes in each of the ten model organisms by DeepLoc (Almagro Armenteros et al. 2017).

• **Table S3** gives the error-corrected number of proteins predicted in each of the seven location classes in each of the ten model organisms by LocTree3 (Goldberg et al. 2014).

• **Fig. S4** shows a racetrack plot of the experimentally determined location from Swiss-Prot (Pundir et al. 2017) and the Human Protein Atlas (Thul et al. 2017) in (A) and the same plot for the prediction of locations for the human proteome through homology based inference in (B).

• **Fig. S5** shows a racetrack plot of the experimentally determined location from Swiss-Prot (Pundir et al. 2017) in (A) and the same plot for the prediction of locations for the yeast proteome through homology based inference in (B).

• **Fig. S6** shows the distance between the location spectra of two reference data sets against the location spectra predicted by different methods before error-correction of the predictions (left) and after error-correction (right).

• **Fig. S7** shows the grouping of the ten reference organisms according to the error-corrected location spectra predicted by DeepLoc.

• **Fig. S8** shows the grouping of the ten reference organisms in UPGMA trees according to the raw and error-corrected location spectra predicted by LocTree2 and according to the raw location spectra predicted by LocTree3 and DeepLoc.

• **Fig. S9** shows the ratio of proteins assigned to each location over the ten reference organisms predicted by LocTree3 in (A) and DeepLoc in (B).

• **Fig. S10** shows the grouping of the ten reference organisms in UPGMA trees according to the location spectra inferred through homology.

• **Fig. S11** shows the grouping of the ten reference organisms in UPGMA trees according to the location spectra if we restrict our analysis only to paralogs or only to homologs for the predictions of LocTree3 in (A) and DeepLoc in (B).

# SOM Tables

Table S1: Conversion of predictions and HPA into seven location classes ^◊^

| *Location class* | *The Human Protein Atlas (HPA) (Thul et al. 2017)* | *MultiLoc2 (Blum et al. 2009)* | *Hum-mPloc3.0 (Zhou et al. 2017)* | *LocTree2 (Goldberg et al. 2012)* | *DeepLoc (Almagro Armenteros et al. 2017)* |
| --- | --- | --- | --- | --- | --- |
| Secreted | N/A | Extracellular | Extracellular | Extracellular | Extracellular |
| Nucleus | nucleoplasm, nuclear bodies, nuclear speckles, nucleus, nucleoli, nucleoli fibrillar centre, nuclear membrane | Nucleus | Nucleus | Nucleus, nucleus membrane | Nucleus |
| Cytoplasm | Cytosol, microtubule, microtubule ends, microtubule organising centre, intermediate filaments, cytoplasmic bodies, actin filaments, centrosome, midbody, cytokinetic bridge, mitotic spindle, rods & rinds, midbody ring | Cytoskeleton, Cytoplasmic | Centriole, Cytoplasm, Cytoskeleton | Cytosol | Cytoplasm |
| Plasma membrane | Plasma membrane, Focal adhesion sites, cell junction | Plasma membrane | Plasma membrane | Plasma membrane | Cell membrane |
| Mitochondrion | Mitochondrion | Mitochondrion | Mitochondrion | Mitochondria, mitochondria membrane, chloroplast^1^, chloroplast membrane^1^, plastid^1^ | Mitochondrion |
| Endoplasmic reticulum | Endoplasmic reticulum | Endoplasmic reticulum | Endoplasmic reticulum | Endoplasmic reticulum, endoplasmic reticulum membrane | Endoplasmic reticulum |
| Golgi apparatus | Golgi apparatus | Golgi apparatus | Golgi apparatus | Golgi apparatus,  Golgi apparatus membrane | Golgi apparatus |

◊ Conversion of the output from the prediction tools (MultiLoc2 (Blum et al. 2009), LocTree2 (Goldberg et al. 2012), Hum-mPloc3.0 (Zhou et al. 2017), Deeploc (Almagro Armenteros et al. 2017)) and from The Human Protein Atlas (Thul et al. 2017) to the seven main location classes used for the comparison of the outputs. For each tool or resource, the mapping from the tool’s output classes to the corresponding reference class is shown. Any entry not fitting in the seven reference classes was excluded from comparison.

1: for LocTree2 (Goldberg et al. 2012) “Chloroplast and plastid classes are valid for plant proteins only, therefore, if the origin of a non-plant protein is known, please consider prediction of these classes as mitochondrial”

Table S2: Location spectra predicted for ten eukaryotes proteomes by DeepLoc ^◊^

| *Organism* | *Secreted* | *Nucleus* | *Cytoplasm* | *Plasma membrane* | *Mitochondrion* | *Endoplasmic reticulum* | *Golgi apparatus* |
| --- | --- | --- | --- | --- | --- | --- | --- |
| HUMAN | 2251 | 5717 | 5722 | 3533 | 1131 | 1370 | 582 |
| ANOGA | 1766 | 2960 | 3094 | 1363 | 882 | 1007 | 335 |
| CAELL | 3133 | 4351 | 4549 | 3442 | 1527 | 1338 | 721 |
| DROME | 2190 | 3295 | 3404 | 1578 | 1024 | 1145 | 408 |
| GORGO | 2060 | 5771 | 5898 | 3234 | 1077 | 1550 | 540 |
| MOUSE | 2479 | 5534 | 5771 | 4466 | 1232 | 1427 | 581 |
| PANTR | 1769 | 5271 | 5274 | 3157 | 1004 | 1338 | 526 |
| RAT | 2082 | 5329 | 5877 | 4216 | 1151 | 1377 | 579 |
| SCHPO | 147 | 1470 | 1785 | 235 | 428 | 621 | 162 |
| YEAST | 375 | 1675 | 2061 | 401 | 612 | 970 | 192 |

◊ Number of proteins predicted in each of the seven sub-cellular localisations (after error correction (Marot-Lassauzaie et al. 2018)) for the ten model organisms by the method DeepLoc (Almagro Armenteros et al. 2017): *Homo sapiens* (HUMAN), *Drosophila melanogaster* (DROME), *Anopheles gambiae* (ANOGA), *Rattus norvegicus* (RAT), *Mus musculus* (MOUSE), *Pan troglodytes* (PANTR), *Gorilla gorilla* (GORGO), *Caenorhabditis elegans* (CAEEL), *Saccharomyces cerevisiae* (YEAST) and *Schizosaccharomyces pombe* (SCHPO).

Table S3: Location spectra predicted for ten eukaryotes proteomes by LocTree3 ^◊^

| *Organism* | *Secreted* | *Nucleus* | *Cytoplasm* | *Plasma membrane* | *Mitochondrion* | *Endoplasmic reticulum* | *Golgi apparatus* |
| --- | --- | --- | --- | --- | --- | --- | --- |
| HUMAN | 2520 | 5686 | 5648 | 3710 | 1174 | 1395 | 656 |
| ANOGA | 1701 | 3128 | 3044 | 1707 | 834 | 864 | 324 |
| CAELL | 2958 | 4818 | 4479 | 2995 | 1714 | 2282 | 542 |
| DROME | 1876 | 3750 | 3377 | 1839 | 962 | 1208 | 435 |
| GORGO | 2585 | 5637 | 5772 | 3495 | 1135 | 1483 | 636 |
| MOUSE | 2693 | 5759 | 5637 | 4651 | 1239 | 1389 | 667 |
| PANTR | 2153 | 5177 | 5201 | 3272 | 1069 | 1300 | 609 |
| RAT | 2315 | 5452 | 5783 | 4417 | 1192 | 1378 | 653 |
| SCHPO | 201 | 1569 | 1571 | 363 | 378 | 784 | 146 |
| YEAST | 625 | 1802 | 1842 | 621 | 422 | 1038 | 178 |

◊ Number of proteins predicted in each of the seven sub-cellular localisations (after error correction (Marot-Lassauzaie et al. 2018)) for the ten model organisms predicted by the method LocTree3 (Goldberg et al. 2014): *Homo sapiens* (HUMAN), *Drosophila melanogaster* (DROME), *Anopheles gambiae* (ANOGA), *Rattus norvegicus* (RAT), *Mus musculus* (MOUSE), *Pan troglodytes* (PANTR), *Gorilla gorilla* (GORGO), *Caenorhabditis elegans* (CAEEL), *Saccharomyces cerevisiae* (YEAST) and *Schizosaccharomyces pombe* (SCHPO).

**Fig. S4**

| **4A:**  **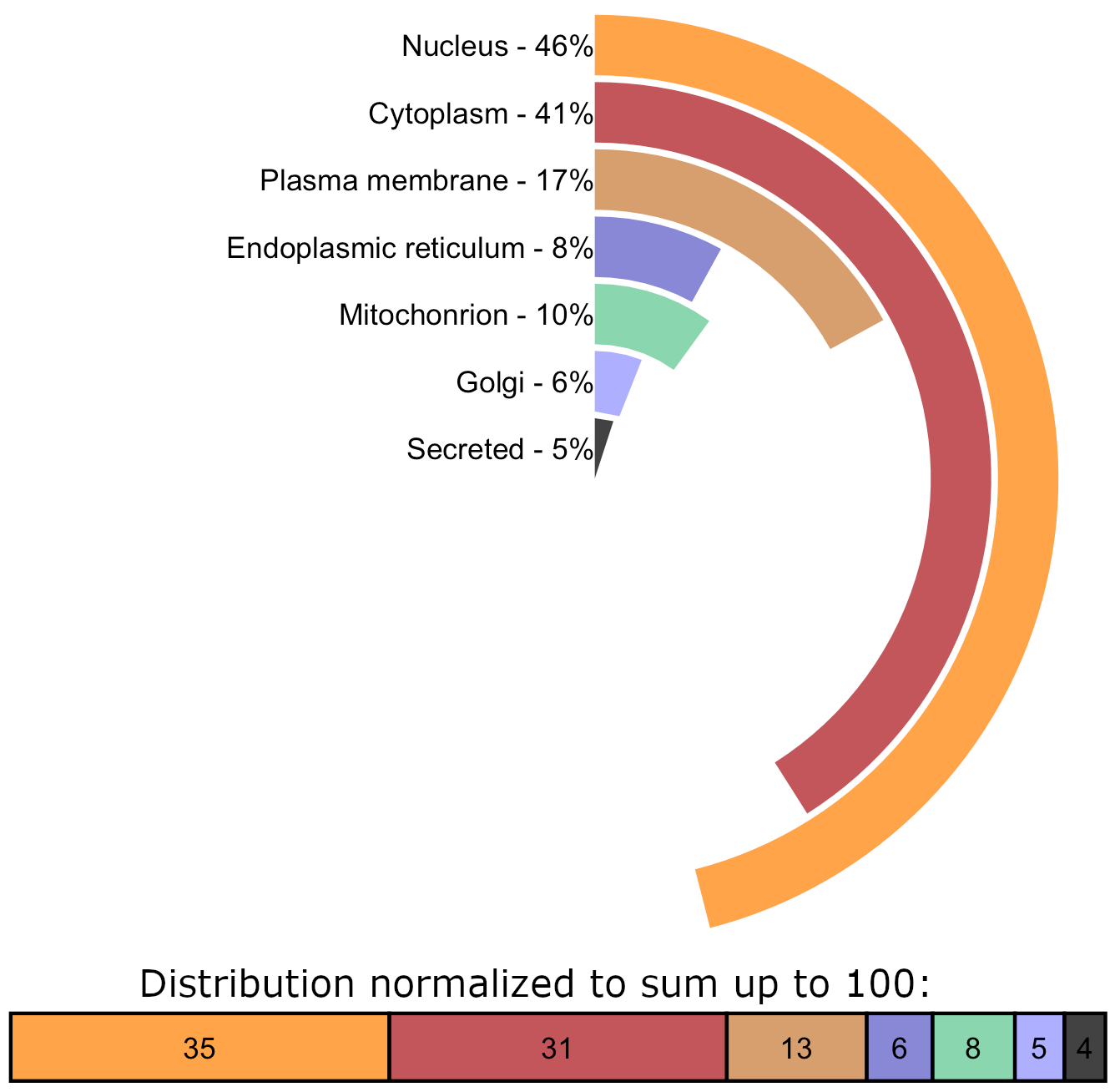** | **4B:**  **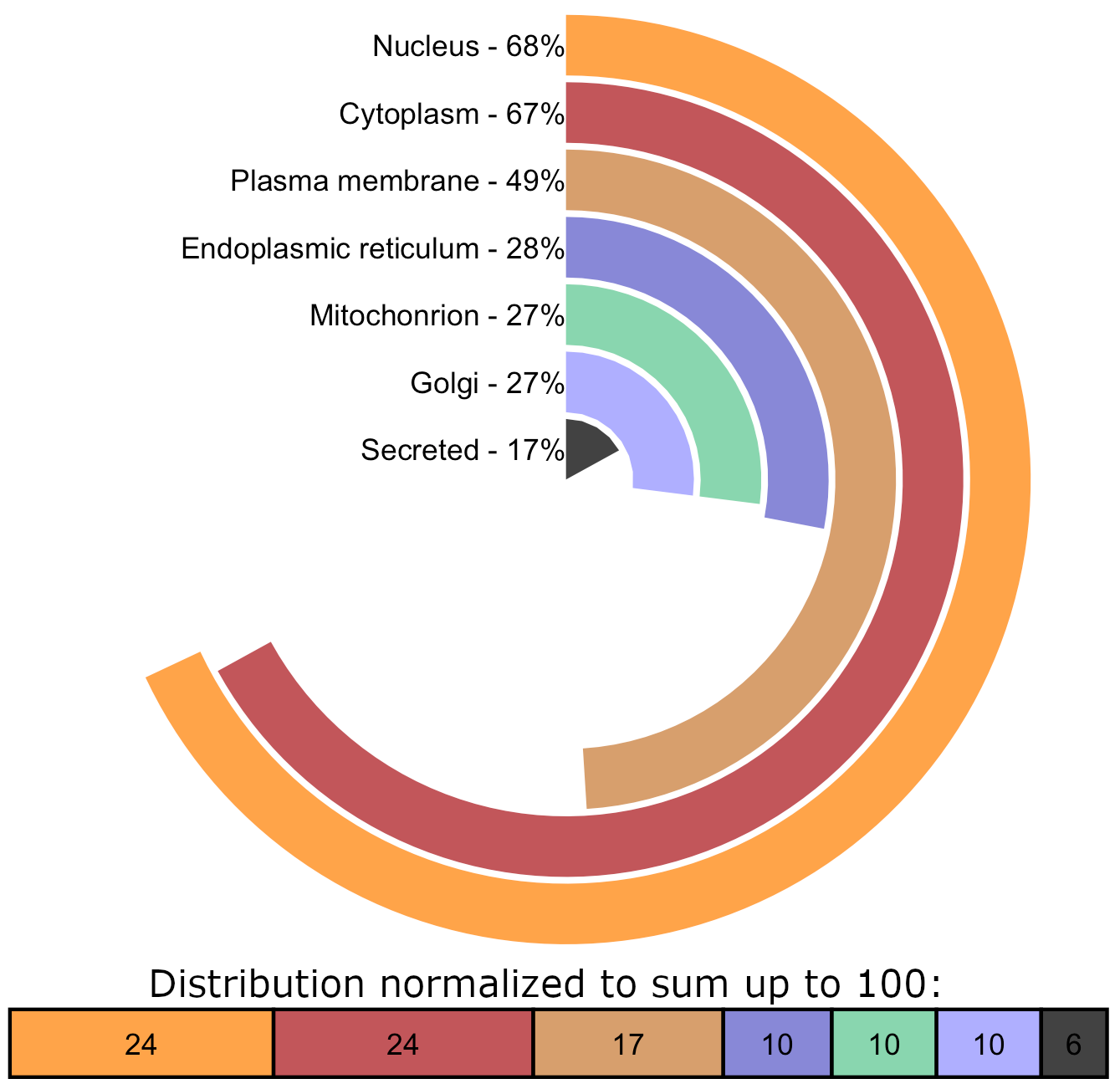** |
| --- | --- |

**Fig. S4:** **Racetrack plot showing the prediction of location for the human proteome from experimental data (A) and through homology based inference (B)**. (A) Distribution of location found for the 7,705 experimentally annotated proteins from the “reliable” HPA annotation or Swiss-Prot. In average, each protein was found in 1.34 compartments. (B) Using UniqueProt (Mika and Rost 2003), we clustered all human proteins at threshold HVAL>4. This resulted in 3,148 families. 1,920 families were covered by experimental annotation in HPA or Swiss-Prot (The UniProt Consortium 2017) from at least one protein in the family. Considering all proteins in a family to have the same location as the annotated proteins of the family, we could infer the location of 18,840 (89%) human proteins. The racetrack plot clearly illustrates limits of such an approach. For instance, it is very unlikely that 68% of all families of human proteins are nuclear, and equally unlikely that the roughly 25% of the human transmembrane proteins constitute 49% of all families, in particular given that most membrane families are unusually large.

**Fig. S5**

| **5A:**  **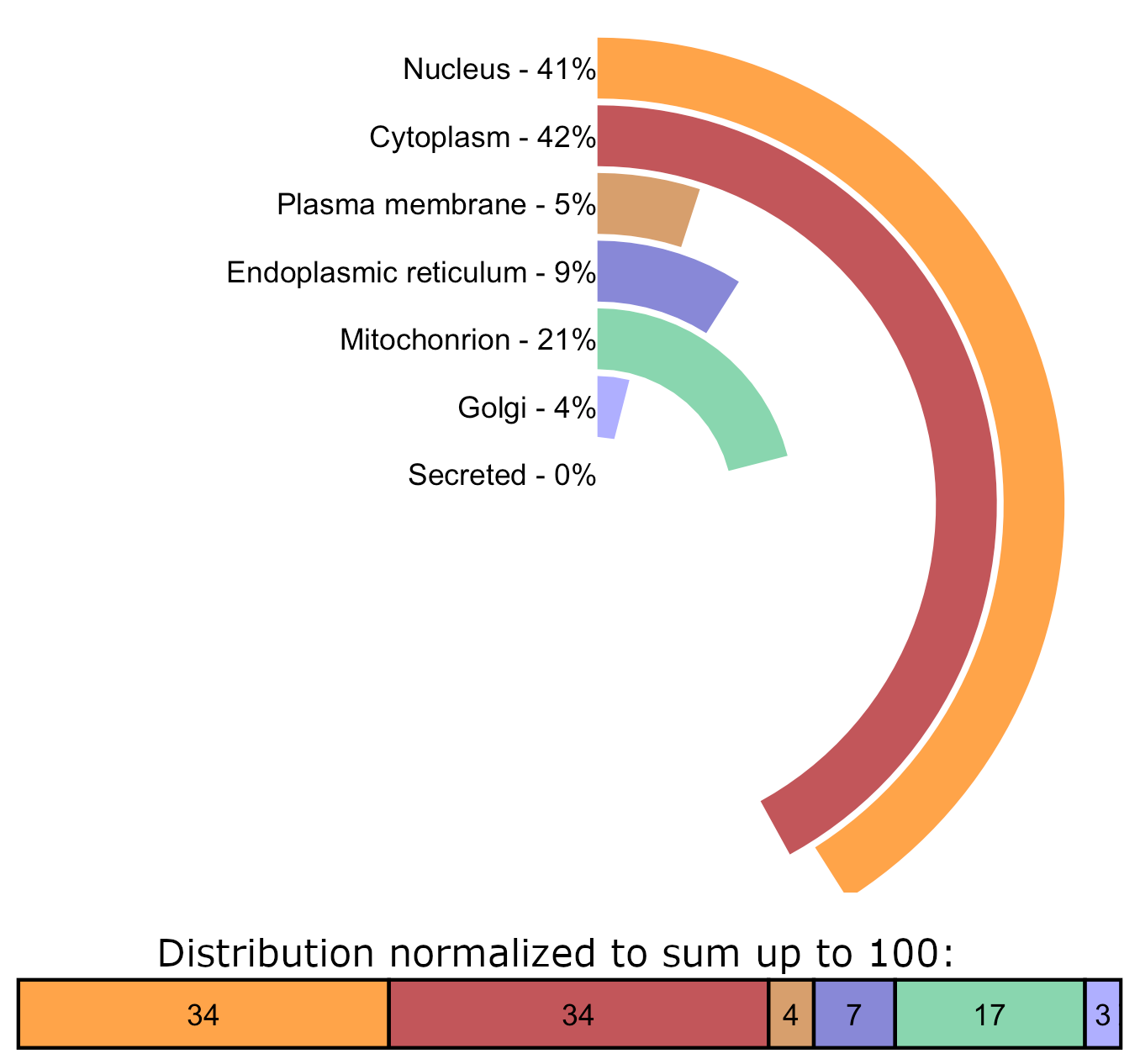** | **5B:**  **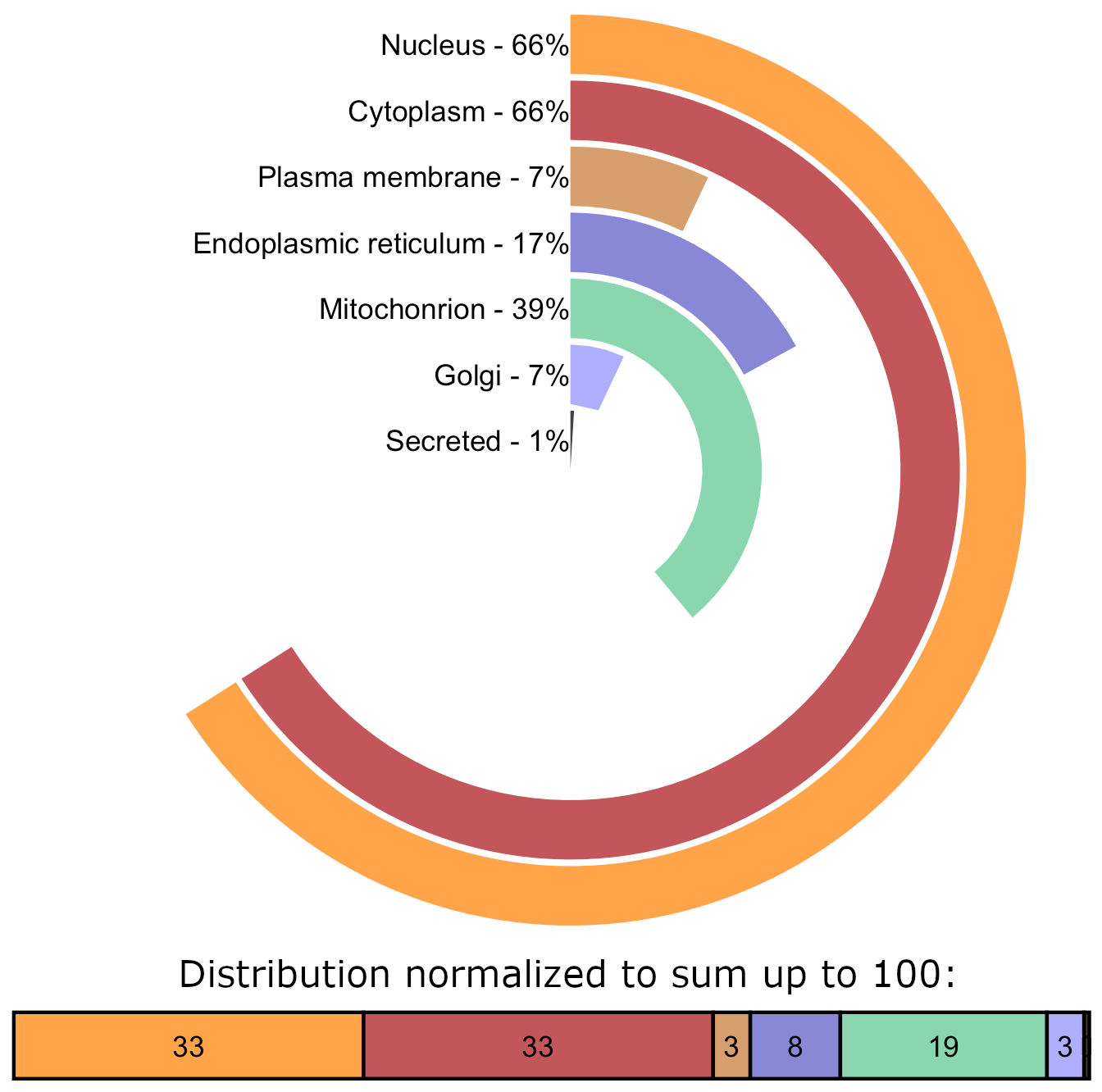** |
| --- | --- |

**Fig. S5: Racetrack plot showing the prediction of location for the yeast proteome from experimental data (A) and through homology based inference (B)**. The colors and ordering of location from Fig. S4 was kept. (A) Distribution of location found for the 2,628 experimentally annotated proteins from Swiss-Prot. In average, each protein was found in 1.22 compartments. (B) Using UniqueProt (Mika and Rost 2003), we clustered all yeast proteins at threshold HVAL>4. The 6,049 proteins were clustered in 1,720 families, and 1,144 were covered by experimental annotation in Swiss-Prot (The UniProt Consortium 2017) from at least one protein in the family. Considering all proteins in a family to have the same location as the annotated proteins of the family, we could infer the location of 5,110 (84%) yeast proteins. This racetrack plot also illustrates limits of such an approach. For instance, it is very unlikely that 66% of all families of yeast proteins are nuclear or cytoplasmic, or that 39% of all families are associated to the mitochondria.

**Fig. S6**


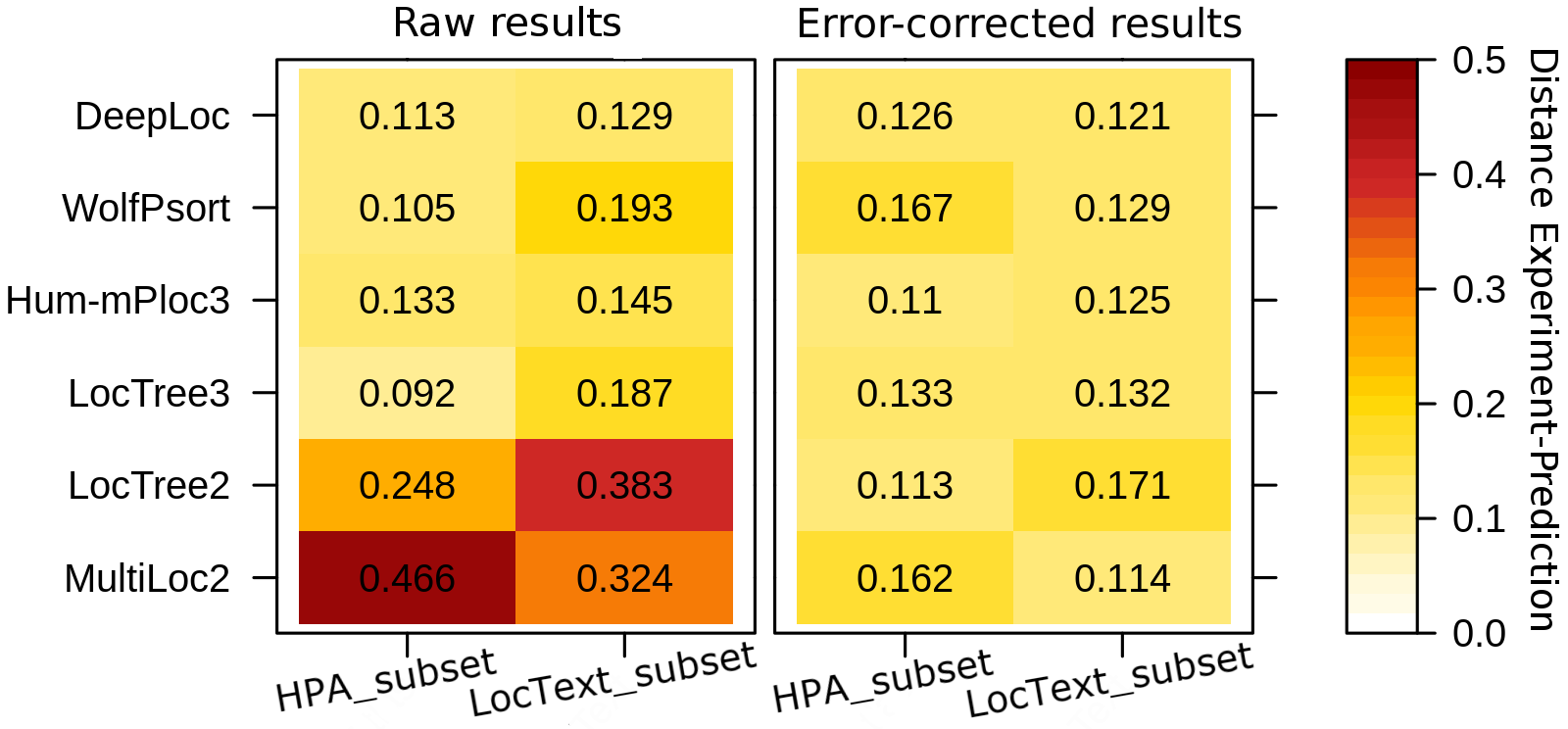


**Fig. S6: Agreement in location spectrum for prediction methods.** For two different data sets of proteins that were not used by the prediction methods (HPA_subset and *LocText_subset*), the heat maps give the Euclidian distances of the location spectra (Eqn. 1) for several prediction methods. The left panel (*Raw results*) show the results directly obtained from the prediction methods, the right panel (*Error-corrected results*) show the same after the application of a simple error-correction using the confusion matrix for each method (Eqn. 2) (Marot-Lassauzaie et al. 2018). A darker, redder color indicates a higher distance, i.e. a worse prediction. Almost all methods improve through the error corrections and almost all estimate the location spectra very accurately.

**Fig. S7**

7A:

**
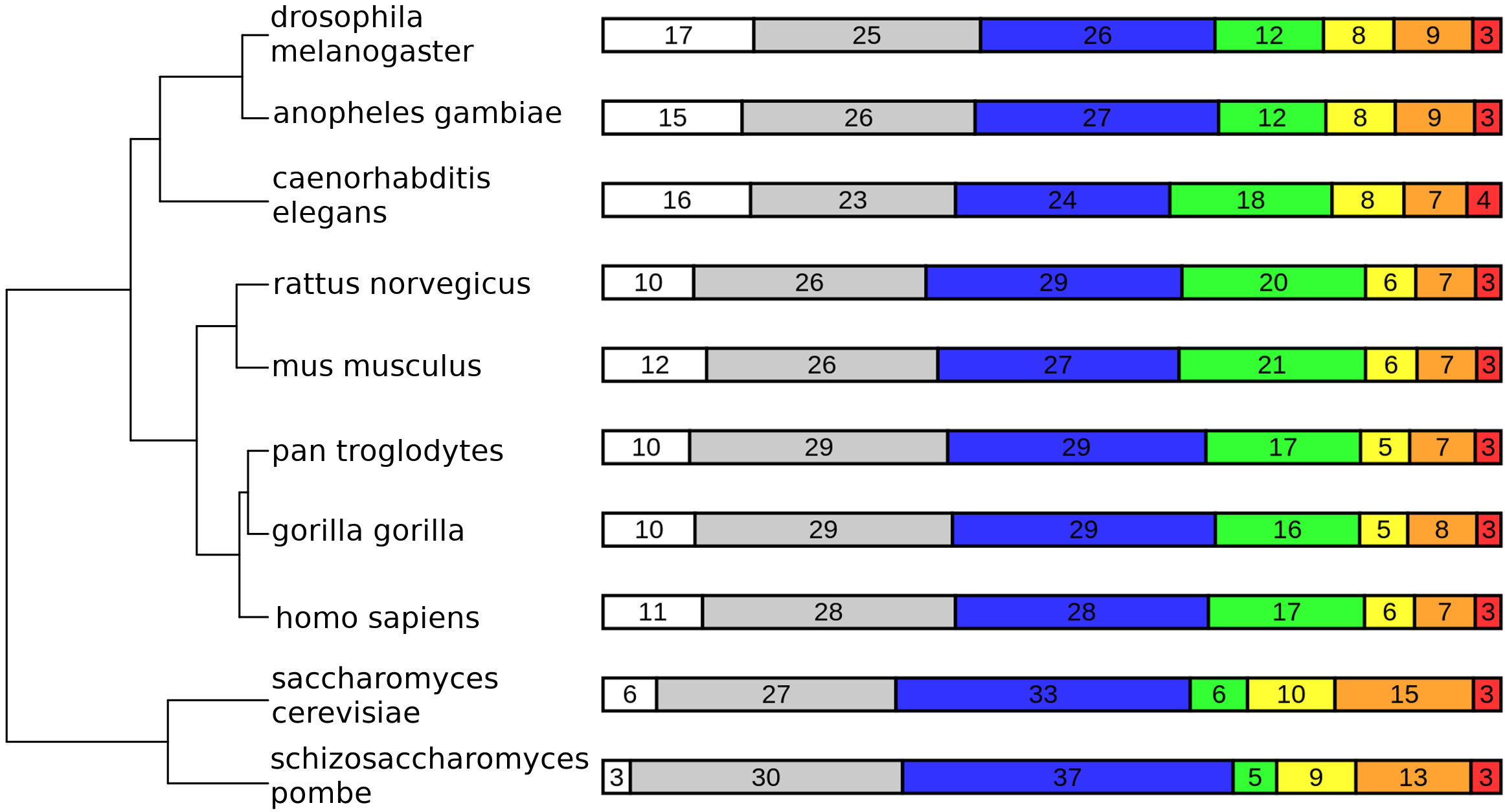
**

7B:

**
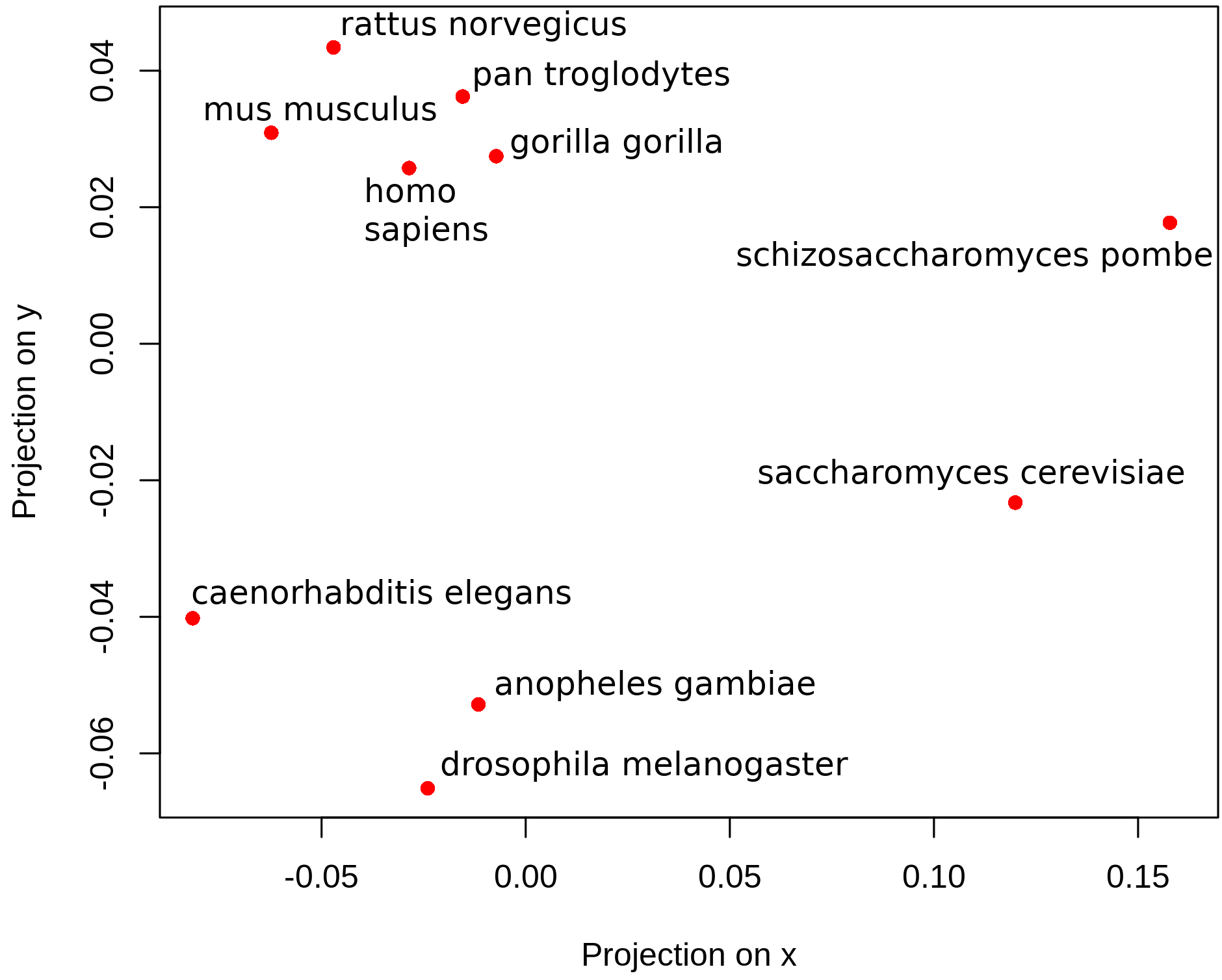
**

|  |
| --- |

**Fig. S7: Grouping of ten eukaryotes according to predicted *location spectra*.** We computed the Euclidean distances between the proteome-wide distributions predicted by *DeepLoc (Almagro Armenteros et al. 2017)* with error-correction (Marot-Lassauzaie et al. 2018) for each of the ten reference organisms. Those values were plotted onto a UPGMA tree (top panel A) and shown through PCA in 2D (lower panel B). **(A)** UPGMA tree built from the predicted distributions from DeepLoc for the 10 organisms along with a bar representing the predicted distribution from LocTree3 in the seven main subcellular location classes is shown for each organism. The seven location classes (from left to right): secreted (white), nuclear (gray), cytoplasmic (blue), plasma membrane (green), mitochondrial (yellow), endoplasmic reticulum (orange) and Golgi apparatus (red). Despite the small differences, the resulting tree largely agrees with what we expect from evolution. **(B)** The PCA adds more details to the comparison between species from the DeepLoc predictions.

**Fig. S8**

| 8A: | 8B: | 8C: |
| --- | --- | --- |
| 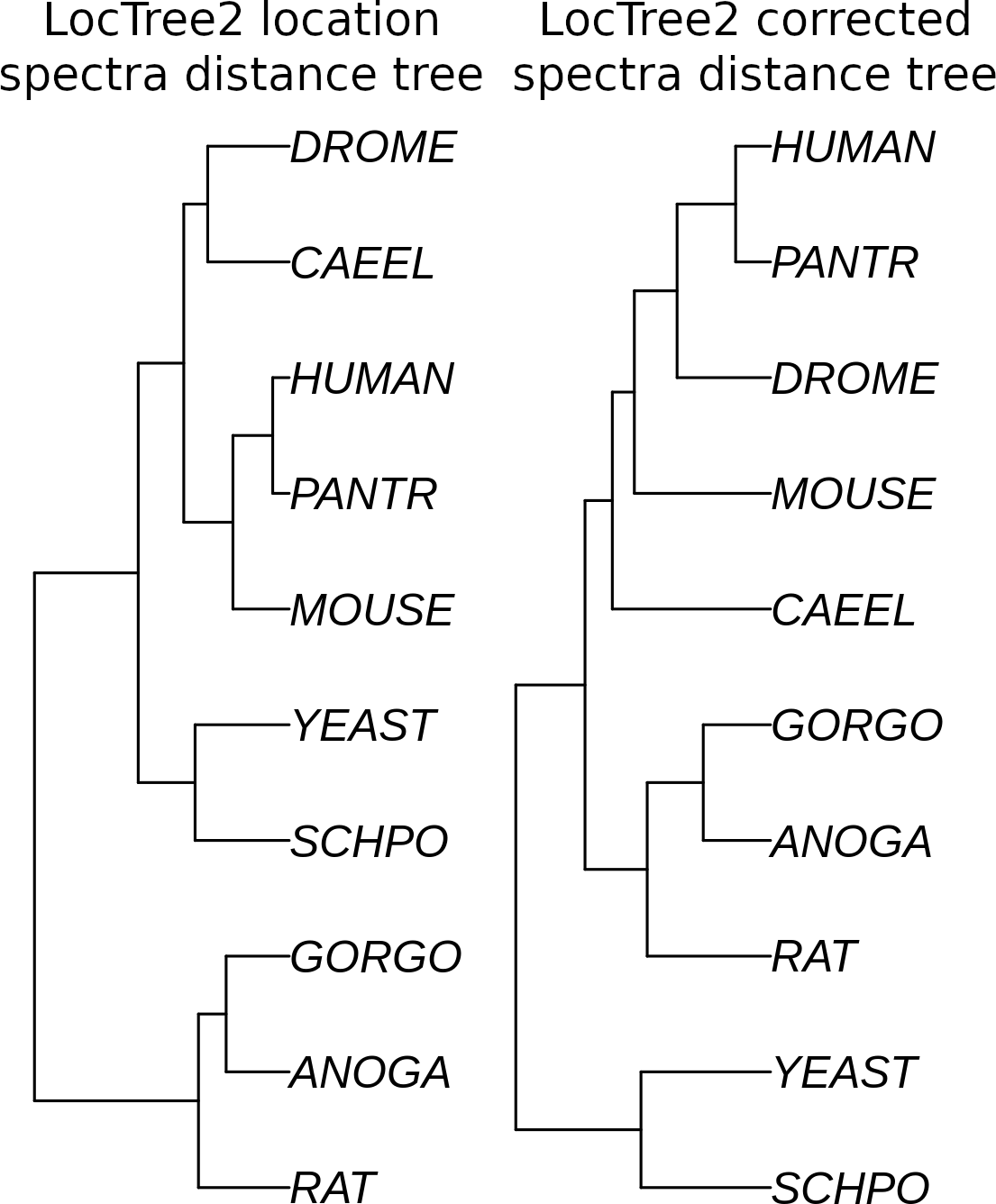 | 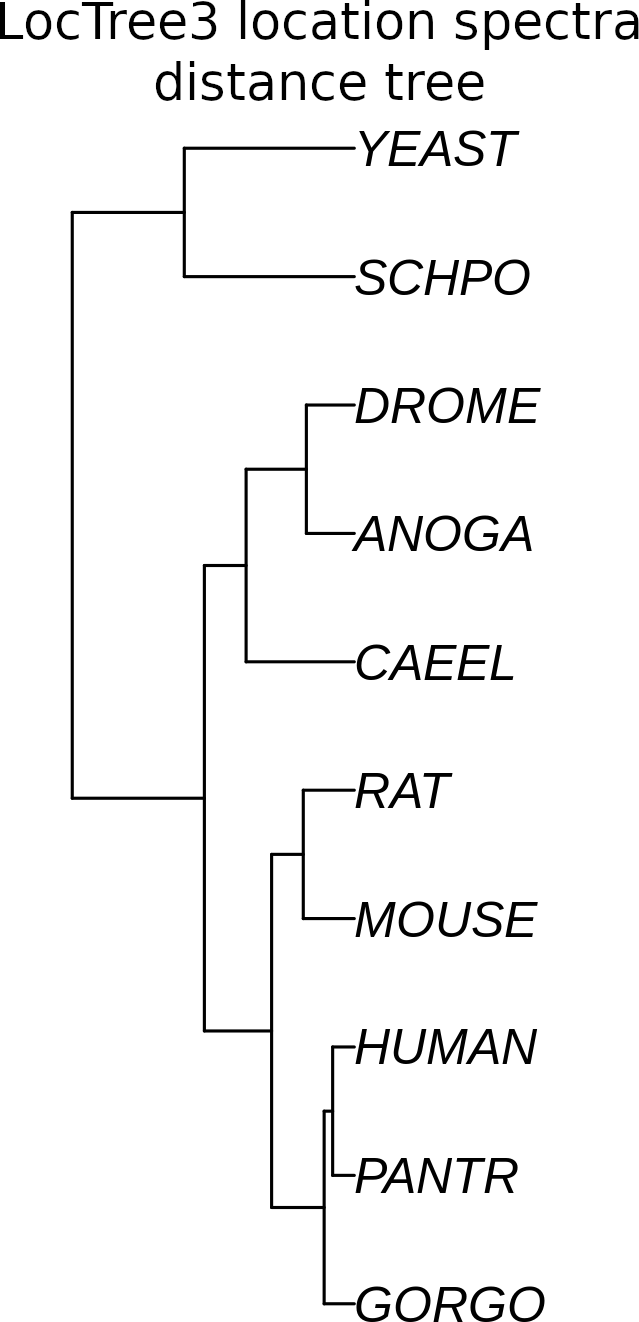 | 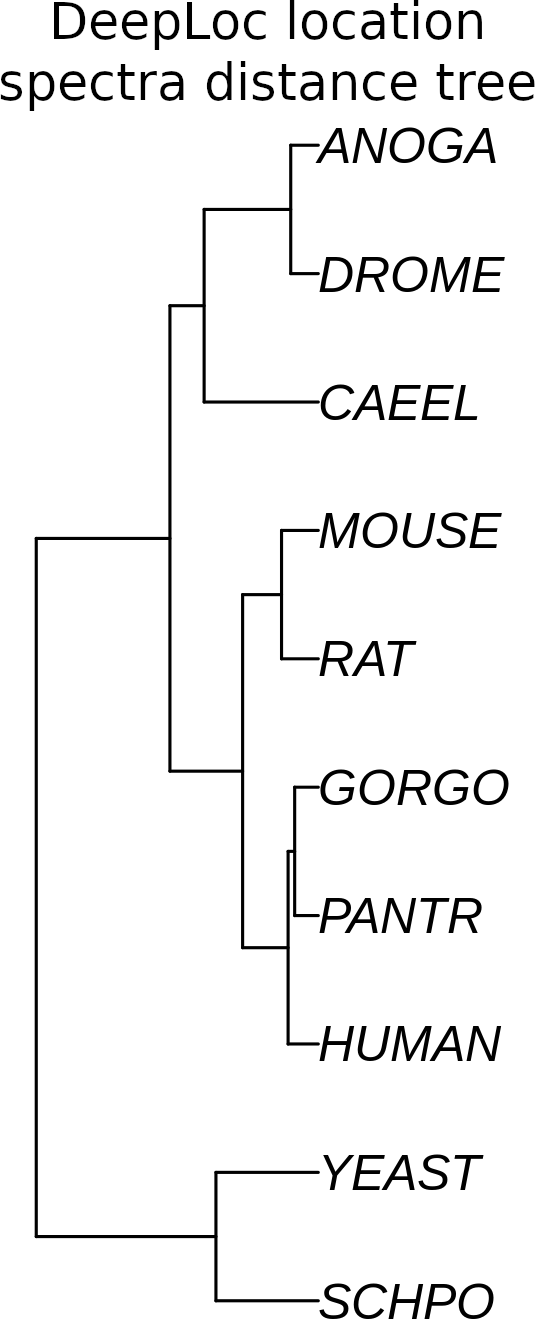 |

|  |
| --- |

**Fig. S8: Grouping of ten eukaryotes according to predicted *location spectra*.** We computed the Euclidean distances between the proteome-wide distributions predicted by LocTree2 (Goldberg et al. 2012), LocTree3 (Goldberg et al. 2014) and DeepLoc (Almagro Armenteros et al. 2017) with and without error-correction (Marot-Lassauzaie et al. 2018) for each of the ten reference organisms. The ten model organisms are abreviated as: *Homo sapiens* (HUMAN), *Drosophila melanogaster* (DROME), *Anopheles gambiae* (ANOGA), *Rattus norvegicus* (RAT), *Mus musculus* (MOUSE), *Pan troglodytes* (PANTR), *Gorilla gorilla* (GORGO), *Caenorhabditis elegans* (CAEEL), *Saccharomyces cerevisiae* (YEAST) and *Schizosaccharomyces pombe* (SCHPO). The values were plotted onto a UPGMA tree for LocTree2 with and without correction (panel A) and for LocTree3 and DeepLoc without correction (panel B and C). **(A)** UPGMA tree built from the raw (left) and error-corrected (right) predicted distributions from LocTree2 for the 10 organisms. The order of the taxonomic units in the tree changes with the correction. **(B)** UPGMA tree built from the raw predicted distributions for LocTree3. **(C)** UPGMA tree built from the raw predicted distributions for DeepLoc. For both LocTree3 and DeepLoc, the order of the taxonomic units does not change with correction.

**Fig. S9**

| 9A DeepLoc:  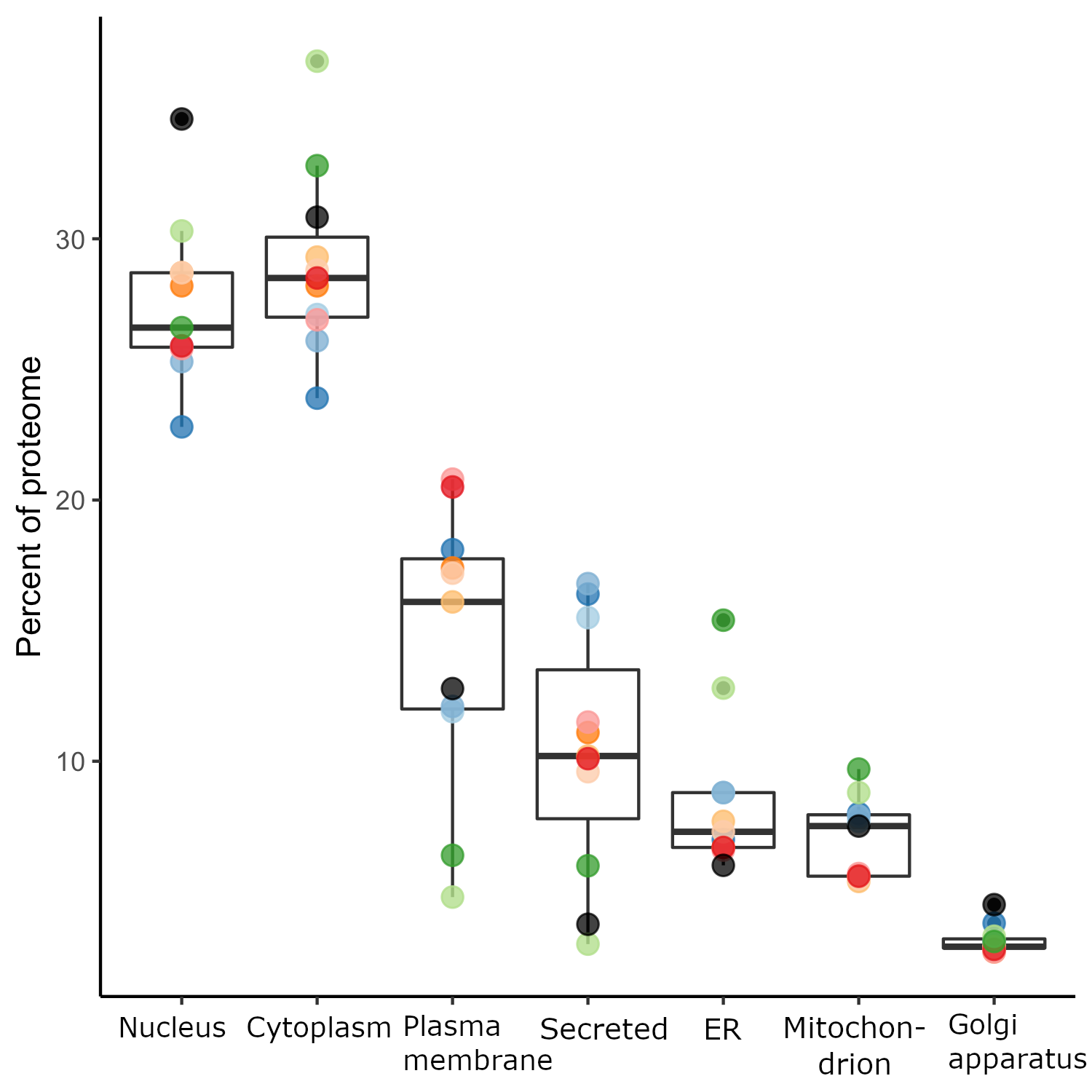 | 9B LocTree3:  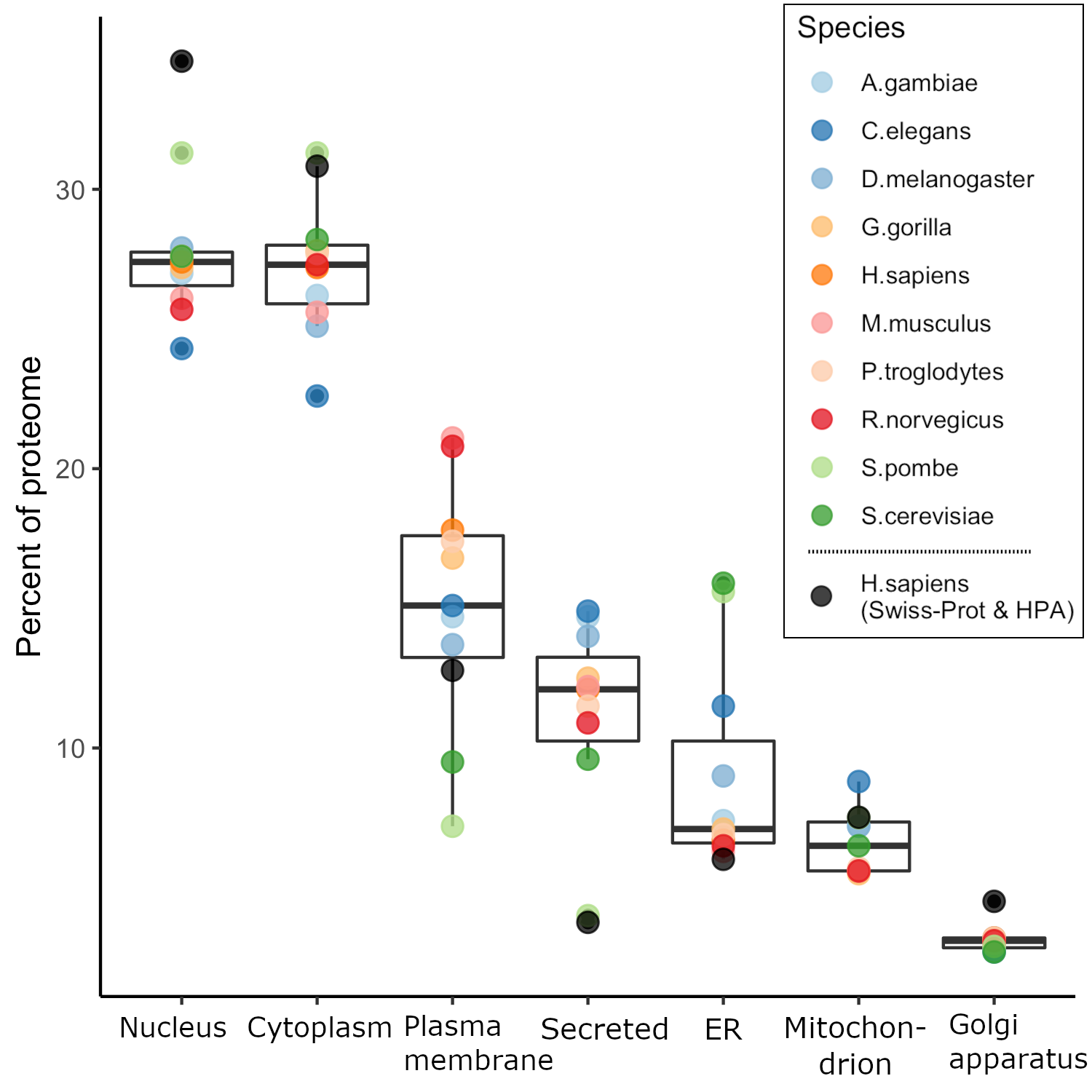 |
| --- | --- |

**Fig. S9: Predicted ratio of location spectra for the 7 main locations over ten reference organisms.** The location spectra predicted by DeepLoc (Almagro Armenteros et al. 2017) (panel A) and LocTree3 (Goldberg et al. 2014) (panel B) were used to get an estimate of the ratio of proteins assigned to each location for the ten reference organisms. The ten model organisms are abbreviated as: *Homo sapiens* (H. sapiens), *Drosophila melanogaster* (D. melanogaster), *Anopheles gambiae* (A. gambiae), *Rattus norvegicus* (R. norvegicus), *Mus musculus* (M. musculus), *Pan troglodytes* (P. troglodytes), *Gorilla gorilla* (G. gorilla), *Caenorhabditis elegans* (C. elegans), *Saccharomyces cerevisiae* (S. cerevisiae) and *Schizosaccharomyces pombe* (S. pombe). The values for the 7,705 human proteins covered by reliable experimental annotation (see fig S4) were plotted in black over the predictions. Only the ratios of the predictions were used to compute the mean and percentiles of the boxplots, the experimental values are shown for visual comparison only. **(A)** Boxplot of the ratio of the proteome assigned to each location as predicted by DeepLoc. **(B)** Boxplot of the ratio of the proteome assigned to each location as predicted by LocTree3. Note that upon first site, you might consider experimental and predicted values to be similar for many classes, for instance, treating extracelluar as a class to ignore due to the experimental challenge of obtaining those data. However, if we excluded extra-cellular altogether, all predicted values would shift up by on average about two percentage points (extra-cellular predicted at about 12%, 6 classes => 12/6~2).

**Fig. S10**

**
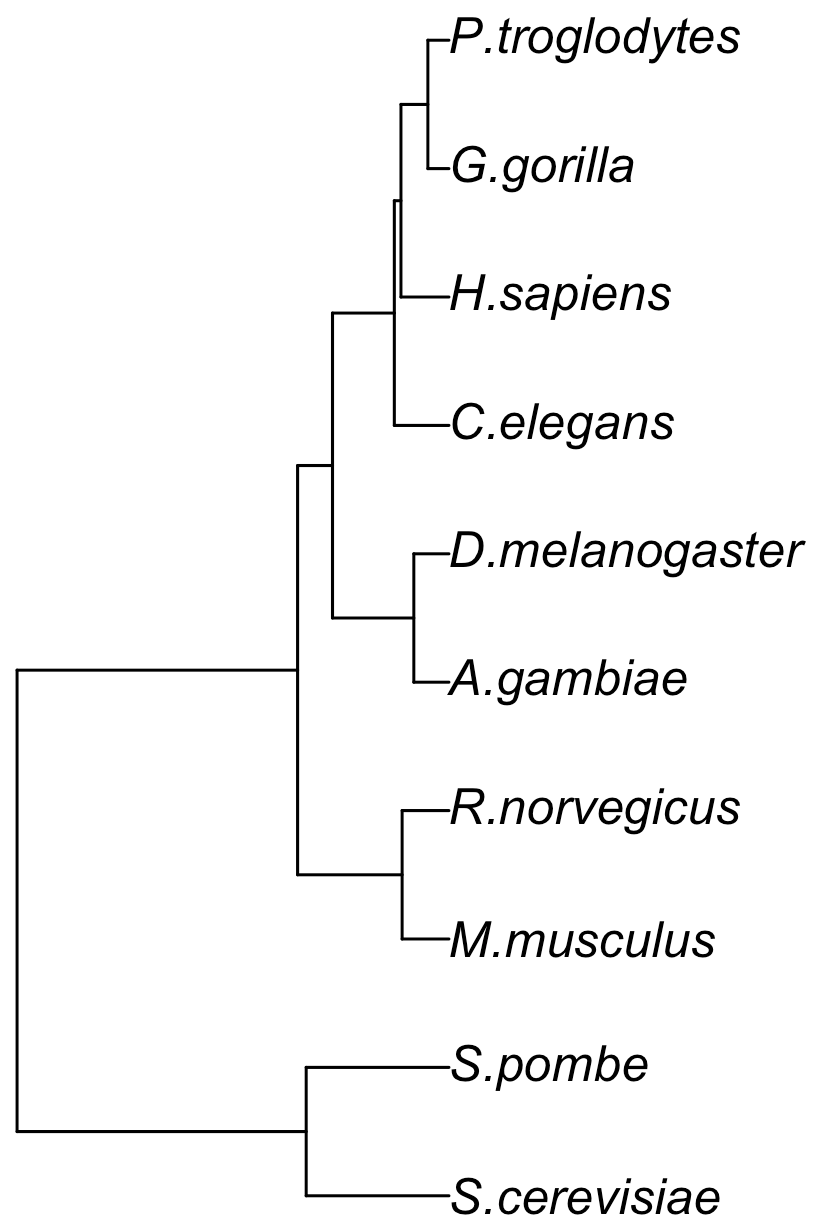
**

**Fig. S10: Grouping of ten eukaryotes according to location spectra inferred from homology.** We used MMseqs2 (Steinegger and Söding 2017) to query the proteomes of each of the ten model organisms against a target reference of proteins with known annotation from Swiss-Prot. The ten model organisms are abbreviated as: *Homo sapiens* (H. sapiens), *Drosophila melanogaster* (D. melanogaster), *Anopheles gambiae* (A. gambiae), *Rattus norvegicus* (R. norvegicus), *Mus musculus* (M. musculus), *Pan troglodytes* (P. troglodytes), *Gorilla gorilla* (G. gorilla), *Caenorhabditis elegans* (C. elegans), *Saccharomyces cerevisiae* (S. cerevisiae) and *Schizosaccharomyces pombe* (S. pombe). The locations of the best match were transferred to the query and used to infer the location spectra for each species. A simple Euclidean distance between the location spectra was used to build a UPGMA tree. While some aspects of this tree reproduce the similarities expected from evolution, s.a. the grouping of the two yeasts, rodents and of the apes, there are a lot of relations that differ from expected. For example, the mammals are not grouped, and the relations between the apes are incorrect.

**Fig. S11**

| 11A:  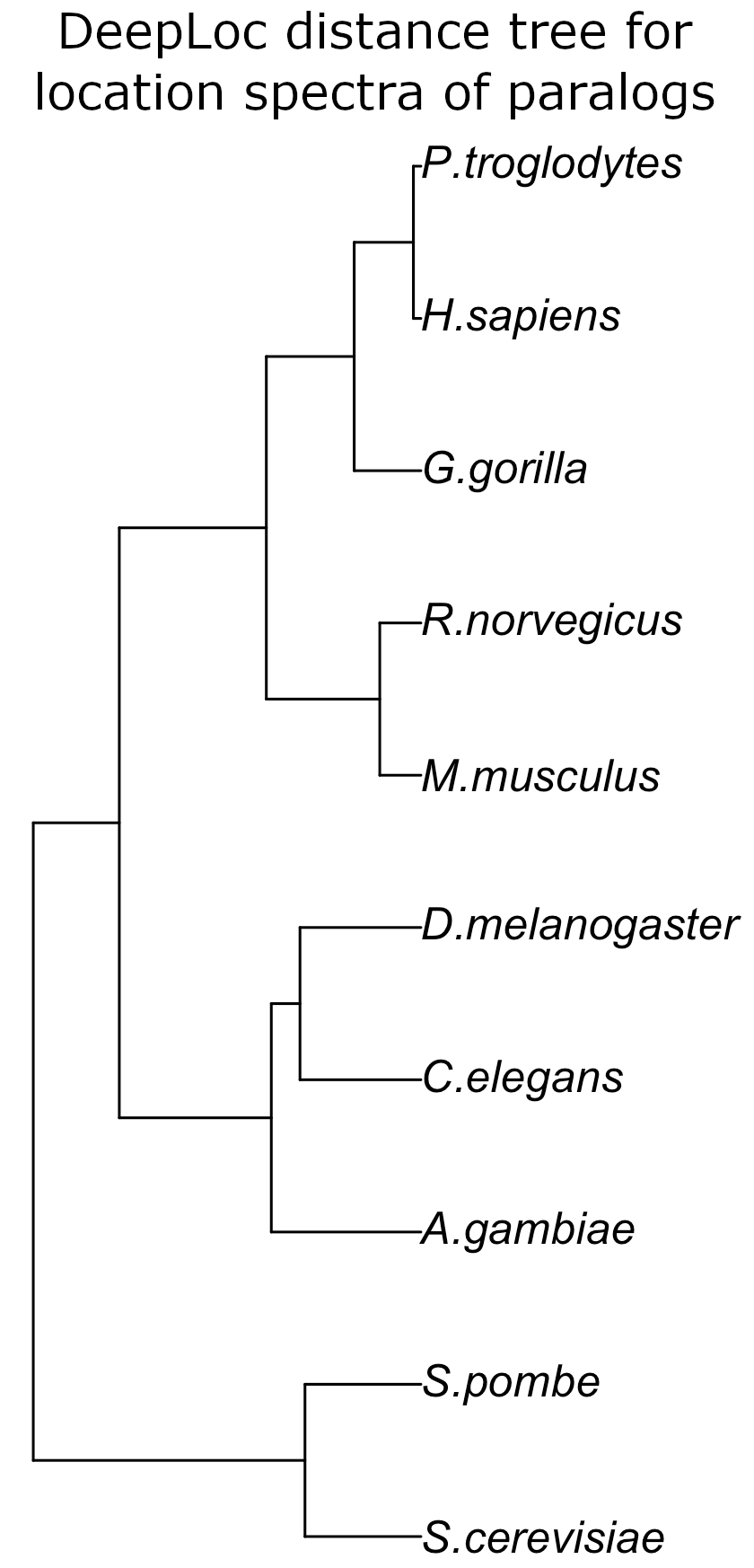 | 11B:  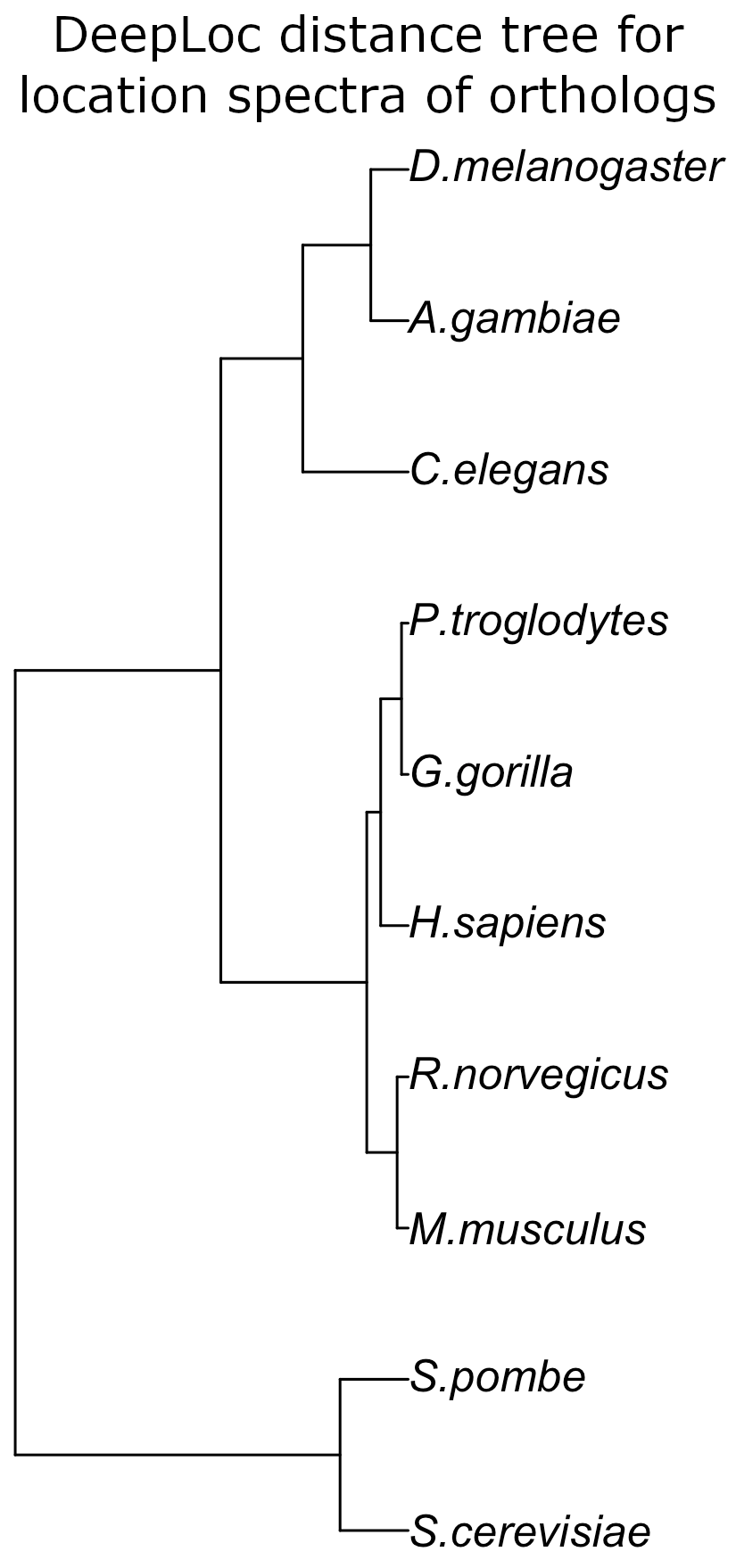 |
| --- | --- |
|  |  |

**Fig. S11: Grouping of ten eukaryotes according to the predicted location spectra for paralogs and orthologs only.** We used InParanoid (Sonnhammer and Östlund 2015) to identify all in-paralogs and orthologs between the species. The ten model organisms are abbreviated as: *Homo sapiens* (H. sapiens), *Drosophila melanogaster* (D. melanogaster), *Anopheles gambiae* (A. gambiae), *Rattus norvegicus* (R. norvegicus), *Mus musculus* (M. musculus), *Pan troglodytes* (P. troglodytes), *Gorilla gorilla* (G. gorilla), *Caenorhabditis elegans* (C. elegans), *Saccharomyces cerevisiae* (S. cerevisiae) and *Schizosaccharomyces pombe* (S. pombe). The predicted location spectra of DeepLoc for paralogs in (A) and orthologs in (B) for the subset of genes were compared through a simple Euclidean distance between the location spectra and used to build the UPGMA tree. The mean distance of location spectra for paralogs is 2.2 times greater for paralogs than for orthologs, but the trees are scaled to remove this effect. The prediction for paralogs reproduces the similarities expected from evolution closer than the ones for orthologs.

References for Supporting Online Material:

Almagro Armenteros JJ, Sønderby CK, Sønderby SK, Nielsen H, Winther O (2017) DeepLoc: prediction of protein subcellular localization using deep learning. Bioinformatics 33:3387

Blum T, Briesemeister S, Kohlbacher O (2009) MultiLoc2: integrating phylogeny and Gene Ontology terms improves subcellular protein localization prediction. BMC Bioinformatics 10:274

Goldberg T, Hamp T, Rost B (2012) LocTree2 predicts localization for all domains of life. Bioinformatics 28:i458

Goldberg T, Hecht M, Hamp T, Karl T, Yachdav G, Ahmed N, Altermann U, Angerer P, Ansorge S, Balasz K, Bernhofer M, Betz A, Cizmadija L, Do KT, Gerke J, Greil R, Joerdens V, Hastreiter M, Hembach K, Herzog M, Kalemanov M, Kluge M, Meier A, Nasir H, Neumaier U, Prade V, Reeb J, Sorokoumov A, Troshani I, Vorberg S, Waldraff S, Zierer J, Nielsen H, Rost B (2014) LocTree3 prediction of localization. Nucleic Acids Res 42:W350

Marot-Lassauzaie V, Bernhofer M, Rost B (2018) Correcting mistakes in predicting distributions. Bioinformatics 34:3385

Mika S, Rost B (2003) UniqueProt: Creating representative protein sequence sets. Nucleic Acids Res 31:3789

Pundir S, Martin MJ, O’Donovan C (2017) UniProt Protein Knowledgebase. In: Wu CH, Arighi CN, Ross KE (eds) Protein Bioinformatics: From Protein Modifications and Networks to Proteomics. Springer New York, New York, NY, pp. 41-55

Sonnhammer EL, Östlund G (2015) InParanoid 8: orthology analysis between 273 proteomes, mostly eukaryotic. Nucleic Acids Res 43:D234

Steinegger M, Söding J (2017) MMseqs2 enables sensitive protein sequence searching for the analysis of massive data sets. Nature Biotechnology 35:1026

The UniProt Consortium (2017) UniProt: the universal protein knowledgebase. Nucleic Acids Res 45:D158

Thul PJ, Akesson L, Wiking M, Mahdessian D, Geladaki A, Ait Blal H, Alm T, Asplund A, Bjork L, Breckels LM, Backstrom A, Danielsson F, Fagerberg L, Fall J, Gatto L, Gnann C, Hober S, Hjelmare M, Johansson F, Lee S, Lindskog C, Mulder J, Mulvey CM, Nilsson P, Oksvold P, Rockberg J, Schutten R, Schwenk JM, Sivertsson A, Sjostedt E, Skogs M, Stadler C, Sullivan DP, Tegel H, Winsnes C, Zhang C, Zwahlen M, Mardinoglu A, Ponten F, von Feilitzen K, Lilley KS, Uhlen M, Lundberg E (2017) A subcellular map of the human proteome. Science 356

Zhou H, Yang Y, Shen HB (2017) Hum-mPLoc 3.0: prediction enhancement of human protein subcellular localization through modeling the hidden correlations of gene ontology and functional domain features. Bioinformatics 33:843
